# Genetic diversity of endosymbiotic bacteria *Wolbachia* infecting two mosquito species of the genus *Eretmapodites* occurring in sympatry in the Comoros archipelago

**DOI:** 10.3389/fmicb.2024.1343917

**Published:** 2024-03-27

**Authors:** Yann Gomard, Sarah Hafsia, Cyrille Lebon, Patrick Rabarison, Ambdoul-bar Idaroussi, Amina Yssouf, Philippe Boussès, Patrick Mavingui, Célestine Atyame

**Affiliations:** ^1^Université de La Réunion, UMR PIMIT (Processus Infectieux en Milieu Insulaire Tropical) CNRS 9192, INSERM 1187, IRD 249, Saint-Denis, île de La Réunion, France; ^2^Service de lutte antivectorielle, ARS Mayotte, Kawéni, France; ^3^National Malaria Control Program, Moroni, Comoros; ^4^UMR MIVEGEC (Maladies Infectieuses et Vecteurs: Écologie, Génétique, Évolution et Contrôle), IRD, CNRS, Université de Montpellier, Montpellier, France

**Keywords:** *Wolbachia*, *Eretmapodites quinquevittatus*, *Eretmapodites subsimplicipes*, mitochondrial genetic diversity, Comoros archipelago

## Abstract

**Introduction:**

The influence of *Wolbachia* on mosquito reproduction and vector competence has led to renewed interest in studying the genetic diversity of these bacteria and the phenotypes they induced in mosquito vectors. In this study, we focused on two species of *Eretmapodites*, namely *Eretmapodites quinquevittatus* and *Eretmapodites subsimplicipes*, from three islands in the Comoros archipelago (in the Southwestern Indian Ocean).

**Methods:**

Using the *COI* gene, we examined the mitochondrial genetic diversity of 879 *Eretmapodites* individuals from 54 sites. Additionally, we investigated the presence and genetic diversity of *Wolbachia* using the *wsp* marker and the diversity of five housekeeping genes commonly used for genotyping through Multiple Locus Sequence Typing (MLST).

**Results and discussion:**

Overall, *Er. quinquevittatus* was the most abundant species in the three surveyed islands and both mosquito species occurred in sympatry in most of the investigated sites. We detected a higher mitochondrial genetic diversity in *Er. quinquevittatus* with 35 reported haplotypes (*N* = 615 specimens, *Hd* = 0.481 and π = 0.002) while 13 haplotypes were found in *Er. subsimplicipes* (*N* = 205 specimens, *Hd* = 0.338 and π = 0.001), this difference is likely due to the bias in sampling size between the two species. We report for the first time the presence of *Wolbachia* in these two *Eretmapodites* species. The prevalence of *Wolbachia* infection varied significantly between species, with a low prevalence recorded in *Er. quinquevittatus* (0.8%, *N* = 5/627) while infection was close to fixation in *Er. subsimplicipes* (87.7%, *N* = 221/252). Both male and female individuals of the two mosquito species appeared to be infected. The analysis of MLST genes revealed the presence of two *Wolbachia* strains corresponding to two new strain types (STs) within the supergroups A and B, which have been named *w*EretA and *w*EretB. These strains were found as mono-infections and are closely related, phylogenetically, to *Wolbachia* strains previously reported in *Drosophila* species. Finally, we demonstrate that maternal transmission of *Wolbachia* is imperfect in *Er. subsimplicipes*, which could explain the presence of a minority of uninfected individuals in the field.

## Introduction

Endosymbiotic bacteria are of increasing interest due to their impact on the biology of arthropods. Some of these bacteria are known to be essential for the evolution of their hosts enabling them to adapt to new ecological niches (Douglas, 1998). Other bacteria provide selective advantages depending on the ecological contexts by providing for example protection against predators (Tsuchida et al., 2010) or pathogens (Oliver et al., 2003; Scarborough et al., 2005; Hedges et al., 2008; Teixeira et al., 2008; Jaenike et al., 2010). In addition to these positive effects, endosymbiotic bacteria are also selfish elements that can manipulate the reproduction of their hosts to increase their own fitness (Duron et al., 2008). This is the case for the bacteria *Wolbachia* which are associated with various reproductive manipulation phenotypes in arthropods (Werren et al., 2008).

*Wolbachia* are maternally inherited alpha-proteobacteria commonly found in arthropods and filarial nematodes (Werren et al., 2008). These bacteria are estimated to be present in up to 66% of insect species (Hilgenboecker et al., 2008; Zug and Hammerstein, 2012; Weinert et al., 2015), thus probably representing the most abundant endosymbiont described to date. *Wolbachia* exibit high genetic diversity and have been classified into 17 phylogenetic groups or supergroups (A to Q) (Baldo et al., 2006; Paraskevopoulos et al., 2006; Bordenstein et al., 2009; Ros et al., 2009; Glowska et al., 2015). The widespread distribution of *Wolbachia* is primarily attributed to their impact on the reproductive biology of their hosts. In arthropods, *Wolbachia* manipulate host reproduction by biasing the sex ratio toward females (the transmitting sex), or by causing sterility through a phenomenon known as cytoplasmic incompatibility (CI) (Werren et al., 2008). Cytoplasmic incompatibility results from sperm-egg incompatibility occuring when *Wolbachia*-infected males mate with either uninfected females or females infected with an incompatible *Wolbachia* strain, resulting in high embryonic mortality reaching up to 100% in certain mosquito species (Laven, 1951; Werren et al., 2008; Atyame et al., 2014). This phenotype is commonly observed in arthropods (Shropshire et al., 2020; Turelli et al., 2022) including in mosquito vectors (Sicard et al., 2019).

Aside from the manipulation of reproduction, *Wolbachia* can also impact the vector competence of mosquitoes, which refers to their ability to become infected with and transmit a pathogen. *Wolbachia* have shown to provide protection against major mosquito-borne pathogens like Dengue virus (DENV), Chikungunya virus (CHIKV) or *Plasmodium* infections (Moreira et al., 2009; Bian et al., 2010, 2013; Hoffmann et al., 2011; Walker et al., 2011; Aliota et al., 2016; Dutra et al., 2016). However, *Wolbachia* can also be linked to increased pathogen transmission in some cases (Hughes et al., 2012; Dodson et al., 2014; Zélé et al., 2014). Because of *Wolbachia*'s influence on both mosquito reproduction and vector competence, these bacteria are increasingly seen as promising tools for mosquito and mosquito-borne diseases control (Bourtzis et al., 2014). In recent years, there has a growing number of studies focusing on the genetic diversity of *Wolbachia* and their associated phenotypes in mosquito vectors (Sicard et al., 2019). *Wolbachia* have been well studied in various medically importance mosquito species of such as *Culex pipiens pipiens* and *Culex pipiens quinquefasciatus* (Duron et al., 2005; Atyame et al., 2011, 2014; Dumas et al., 2013), *Aedes albopictus* (Kambhampati et al., 1993; Armbruster et al., 2003; Tortosa et al., 2010; Zouache et al., 2011), as well as more recently in *Anopheles* species (Baldini et al., 2014; Ayala et al., 2019) and *Aedes aegypti* (Coon et al., 2016; Thongsripong et al., 2018). However, there have been limited studies on the presence of *Wolbachia* in mosquitoes of the *Eretmapodites* genus (Tokash-Peters et al., 2022; Osuna et al., 2023), despite their role in the arbovirus transmission (arthropod-borne viruses) (Bamou et al., 2021; Cêtre-Sossah et al., 2023).

Mosquitoes from the *Eretmapodites* genus (Theobald, 1901) (subfamily: Culicinae; tribe: Aedini) are exclusively Afrotropical species occurring in continental Africa (Harbach, 2007), Madagascar (Tantely et al., 2016), and in the islands of the Comoros archipelago (composed of four volcanic islands: Grande Comore, Mohéli, Anjouan and Mayotte) within the Southwestern Indian Ocean (Le Goff et al., 2014; Boussès et al., 2018). A total of 51 *Eretmapodites* species have been described so far (https://mosquito-taxonomic-inventory, accessed in November 2023) (Harbach, 2013), most (32 species) from Cameroon (Bamou et al., 2021), while only four species are known in Madagascar (Tantely et al., 2016), and two species are reported in the Comoros archipelago (Le Goff et al., 2014; Boussès et al., 2018). *Eretmapodites* species are mostly found in forested areas but some species are also adapted to rural and peri-urban environments (Le Goff et al., 2014; Boussès et al., 2018; Bamou et al., 2021). Along with their aggressive daytime biting behavior, these mosquitoes are known to bite both animals and humans (Musa et al., 2020); as a result they have the potential to serve as bridge vectors of pathogens between animals and humans. Different arboviruses such as Rift Valley fever virus (RVFV), Semliki Forest virus (SFV), or CHIKV have been detected and/or isolated from *Eretmapodites* mosquitoes [review in Bamou et al. (2021)]. In addition, some studies have described the ability of *Eretmapodites* mosquitoes to transmit arboviruses under laboratory conditions. For example, Bauer (1928) showed that *Eretmopodites chrysogaster* is able to transmit the Yellow Fever virus (YFV). The study of Mclntosh and coworkers (McIntosh et al., 1980) demonstrated the ability of *Eretmapodites quinquevittatus* to transmit the RVFV. Likewise, it has been recently shown that *Eretmapodites subsimplicipes* is a competent vector for the transmission of RVFV (Cêtre-Sossah et al., 2023). Despite the medical interest of *Eretmapodites* species, the biology, ecology and genetics of these mosquitoes remain poorly investigated.

In this study we focused on two *Eretmapodites* species, namely *Er. quinquevittatus* and *Er. subsimplicipes*, from three islands in the Comoros archipelago: Grande Comore, Mohéli and Mayotte. We used molecular identification to determine the abundance of each mosquito species on the surveyed islands. Then, we examined the presence of *Wolbachia* in both *Eretmapodites* species through the presence/absence of the *Wolbachia* surface protein gene *wsp* (Braig et al., 1998). The genetic diversity of the detected *Wolbachia* was further characterized by sequencing the *wsp* gene and the five housekeeping genes developed for the *Wolbachia* typing (MLST) (Baldo et al., 2006). Finally, we examined the vertical transmission of *Wolbachia* in a laboratory colony of *Er. subsimplicipes*. The role of *Wolbachia* in the evolution of *Eretmapodites* species is discussed.

## Materials and methods

### Mosquito sampling

**Figure 1:**
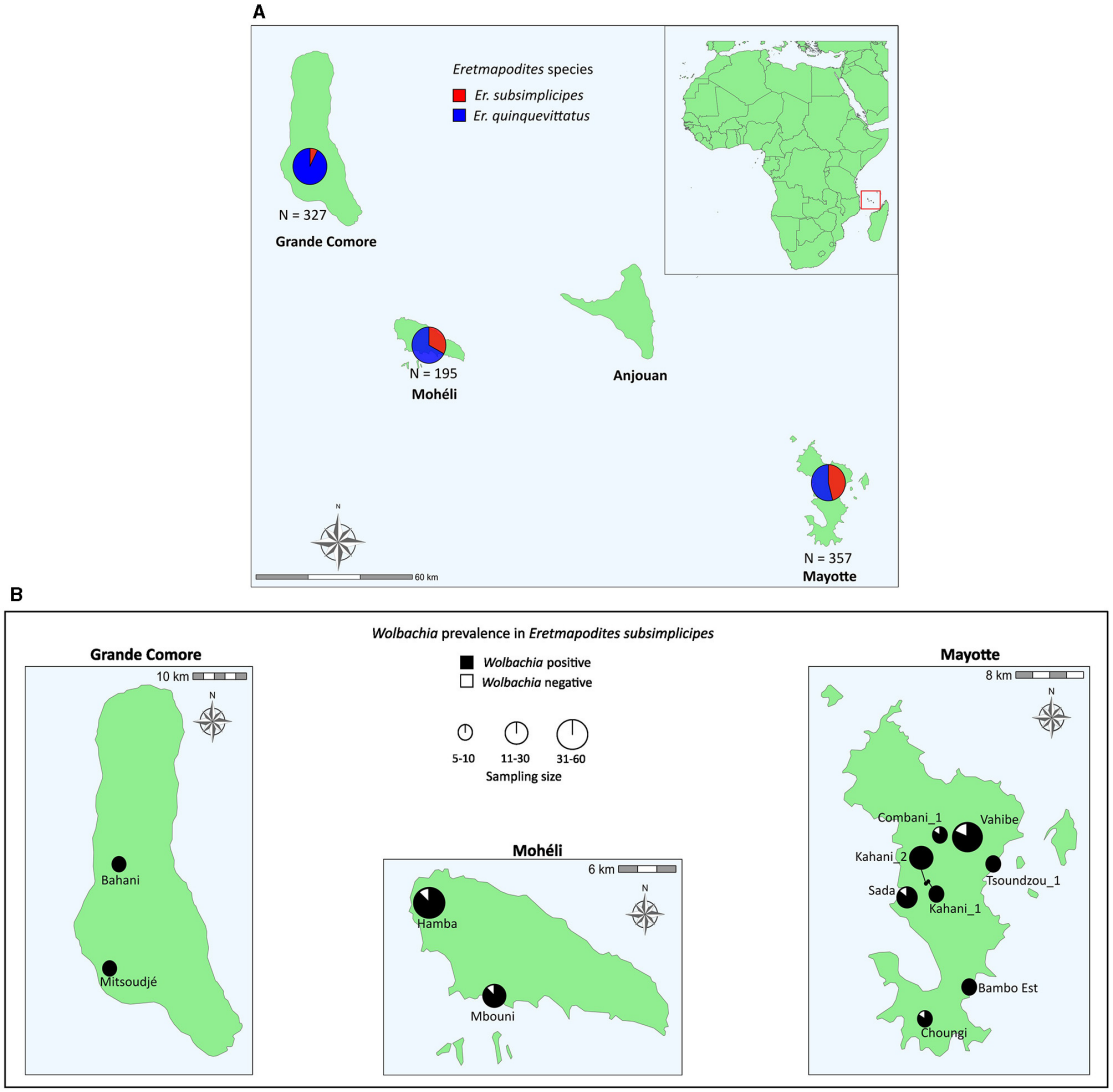
Maps showing the abundance of *Eretmapodites quinquevittatus* and *Eretmapodites subsimplicipes* **(A)** and the prevalence of *Wolbachia* in field populations of *Er. subsimplicipes* **(B)** in three islands of the Comoros archipelago (Grande Comore, Mohéli and Mayotte). Mosquitoes were sampled as adults and identified with the sequencing of the mitochondrial *COI* gene. The prevalence of *Wolbachia* is based on the presence/absence of the *wsp* gene. Only sites with more than five individuals are shown (see Supplementary Table 1 for more details).

Adult *Eretmapodites* specimens were collected in 2019 (March to May and November to December) from 54 natural breeding sites on three islands of the Comoros archipelago (in the Southwestern Indian Ocean): Grande Comore (18 sites), Mohéli (eight sites), and Mayotte (28 sites) (Figure 1; Supplementary Table 1). Larvae were also collected in the site Bambo Est on Mayotte in March 2019 and March 2022 to establish a laboratory colony for testing vertical transmission of *Wolbachia* (see below). Adult mosquitoes were captured using portable electric aspirators (BioQuip InsectaVac aspirator, Bioquip, CA). Collected adults were introduced in cages (16 × 16 × 16 cm) and brought to the laboratory where they were sorted by sex and individually stored in 1.5 ml tubes at −80°C (for samples from Mayotte) or in 70% ethanol (for samples from Grande Comore and Mohéli) until morphological identification and molecular analyses.

### Mosquito identification

The larvae and adults of *Eretmapodites* from the Bambo Est site (Mayotte) and collected in March 2019 and March 2022 were identified morphologically using taxonomic keys (Edwards, 1941; Hopkins, 1952; Service, 1990). The larvae of *Er. quinquevittatus* have a characteristic thick, heavily chitinized 3-VIII seta and, in adults, the scutum is decorated with five parallel bands of dark scales on a yellow-brown background. The larvae of *Er. subsimplicipes* are easily distinguished from those of *Er. quinquevittatus* by lateral setae of abdominal segments I-VI inserted on a sclerotized conical tubercle and, in adults, a more homogeneous scutum devoid of such dark bands. Molecular identification of species was realized on larvae and all collected adult specimens through PCR amplification and sequencing of a 658 bp fragment of the mitochondrial cytochrome c oxidase subunit 1 encoding gene (*COI*) (Folmer et al., 1994) (primers listed in Supplementary Table 2). DNA extraction, PCR, and sequencing were performed as described below.

### *Wolbachia* genotyping

*Wolbachia* detection was performed in all sampled adults using PCR targeting the surface protein gene *wsp* (Braig et al., 1998) (Supplementary Table 2) which is more variable than the slowly evolving *16S rRNA* gene (Zhou et al., 1998). For *wsp*-positive samples, *Wolbachia* were genotyped by sequencing the *wsp* gene and the five MLST loci: *coxA, fbpA, ftsZ, gatB* and *hcpA* (Baldo et al., 2006) (Supplementary Table 2). For all individuals in which *Wolbachia* DNA could not be amplified, the quality of the DNA template was checked by the amplification of the *COI* gene.

### PCR amplification and sequencing

DNA was extracted from individual mosquitoes using the QIAcube HT robotic workstation and the associated Cador Pathogen 96 QIAcube HT Kit (Qiagen) following manufacturer's recommendations, eluted with 100 μl AVE buffer (Qiagen) and eventually stored at −20°C until molecular investigations. PCRs were performed with 0.5 ng of genomic DNA in a 25 μl final volume reaction containing 8.5 μl of water, 12.5 μl of GoTaq^®^ G2 HotStart Green Master Mix (Promega), and 1 μl of each primer (10 μM) (Supplementary Table 2). All PCR programs included an initial denaturation step at 95°C for 5 min, followed by 36 cycles (30 cycles for the *COI* gene) at 94°C for 30 s, 52°C−59°C for 60 s and 72°C for 90 s, and a final elongation step at 72°C for 7 min. Amplified DNA fragments were ran on 1.5% agarose gel electrophoresis stained with 1X GelRed^TM^ (Biotium Inc.) and visualized under ultraviolet light. PCR products were Sanger sequenced on both strands (Genoscreen, Lille, France). Only unique generated sequences were submitted to GenBank under the following accession numbers: OR282837-OR282884, OR296528-OR296530, OR296531-OR296533, OR296534-OR296536, OR296537-OR296539, OR296540-OR296543, and OR296544-OR296547 for *COI, coxA, fbpA, ftsZ, gatB, hcpA*, and *wsp*, respectively.

### Sequences

All sequences were visually inspected and manually edited using Geneious Prime v.2022.2.2 (Kearse et al., 2012). For the *COI* gene, comparisons with public sequences were performed using basic local alignment search tool (BLAST) (www.ncbi.nlm.nih.gov/BLAST, accessed on 28 July 2023) from GenBank. The mitochondrial haplotype diversity (*Hd*) and nucleotide diversity (π) were calculated in the software DnaSP v6.12.03 (Rozas et al., 2017). For *Wolbachia* genes, the generated sequences were compared with data available in the *Wolbachia* MLST database (https://pubmlst.org/organisms/wolbachia-spp, accessed on 03 August 2023) (Jolley et al., 2018). For each MLST gene, a new allele was considered if there was at least one nucleotide difference with alleles already present in the pubMLST database. Thereafter, the combination of alleles allowed identifying the Sequence Types (STs) among those existing or to propose new STs.

### Phylogenetic analysis

Phylogenetic relationships were evaluated for *Eretmapodites COI* and *Wolbachia* genes. Only unique mitochondrial haplotypes and bacterial alleles were included in the analyses. For *Wolbachia*, phylogenetic analyses were conducted for each of the six sequenced genes and on all five MLST concatenated genes (*coxA, fbpA, ftsZ, gatB*, and *hcpA*, in this order). The phylogenies were constructed with data from Baldo et al. (2006). For each data set, the best-fitting model of sequence evolution was determined using jMoldelTest v.2.1.4 (Darriba et al., 2012). Then, phylogenetic constructions were performed using MrBayes v.3.2.3 (Ronquist et al., 2012). For each phylogeny, the analysis corresponded to two independent runs of four incrementally heated Metropolis Coupled Markov Chain Monte Carlo (MCMCMC) starting from a random tree. The MCMCMC was run for 10 million generations with trees and associated model parameters sampled every 100 generations. The convergence level was accessed with an average standard deviation of split frequencies inferior to 0.05. The 10% initial trees for each run were discarded as burn-in and the phylogeny along with posterior probabilities were obtained from the remaining trees. The resulting Bayesian phylogeny trees were visualized and annotated with FigTree v.1.4.2 (Rambaut, 2014).

### Vertical transmission of *Wolbachia*

Since only the *Er. subsimplicipes* species could be reared from larvae collected in the Bambo Est site (Mayotte) in March 2019, we used it to examine the vertical transmission of *Wolbachia*. Field larvae (F_0_ generation) were brought to the laboratory and kept alive in the insectary where they were identified morphologically and maintained under standard rearing conditions (27°C and 80% relative humidity with a 12h:12h photoperiod). Larvae were supplied every 2 days with yeast tablets and adults were fed with 10% sucrose solution. To get eggs and ensure the maintenance of mosquitoes, females were blood-fed using a Hemotek feeding system (Hemotek Limited, GreatHarwood, UK) with defibrinated cow blood. The eggs of the next generation (F_1_ generation) were collected and reared to adulthood. The amplification process was performed over four generations (F_4_ generation) to increase the number of females for the experiment. Then, the vertical transmission of *Wolbachia* was assessed using females and males from the established laboratory colony. Females of the F_4_ generation were allowed to mate in the laboratory with males from the same colony. After mating, the females were blood-fed and individually isolated to lay eggs. Then, the presence of *Wolbachia* was tested for each female by PCR using the *wsp* gene as described above. The offsprings from each *Wolbachia*-infected female were kept alive until adulthood and males and females were screened for the presence of *Wolbachia*.

## Results

### *Eretmapodites quinquevittatus* is more abundant than *Eretmapodites subsimplicipes*

Larvae and adults collected in the site Bambo Est (Mayotte) in March 2019 and March 2022 were morphologically identified as *Er. subsimplicipes*. *COI* sequencing of these morphologically identified specimens showed a closed match with the published sequence of *Er. subsimplicipes* from Mozambique (GenBank accession number: LC664011, 99.8%−100.0% percentage of identity based on 633 bp), thus confirming the identification of our specimens. We then sequenced a total of 879 mosquitoes (655 females and 224 males) from Grande Comore (*N* = 327), Mohéli (*N* = 195), and Mayotte (*N* = 357). The comparison of the obtained *COI* sequences with the GenBank database indicated the presence of two *Eretmapodites* species: *Er. quinquevittatus* (GenBank accession number: LC664009, 98.4%−100.0% percentage of identity based on 629 bp) and *Er. subsimplicipes* (GenBank accession number: LC664011, 99.7%−100.0% percentage of identity based on 633 bp). Among the sequences, 71.3% (*N* = 627/879) and 28.7% (*N* = 252/879) belonged to *Er. quinquevittatus* and *Er. subsimplicipes*, respectively (Supplementary Tables 1, 3). *Eretmapodites quinquevittatus* appeared more common than *Er. subsimplicipes* in all three investigated islands (Figure 1A). Both *Eretmapodites* species were found in sympatry in 31 out of 54 sampled sites (seven sites in Grande Comore, seven sites in Mohéli and 17 sites in Mayotte), while *Er. quinquevittatus* was found alone in 22 sites (11 sites in Grande Comore, one site in Mohéli and ten sites in Mayotte) and *Er. subsimplicipes* alone at one site (Bambo Est) on Mayotte (Supplementary Table 1).

### Higher mtDNA polymorphism in *Er. quinquevittatus*

**Figure 2:**
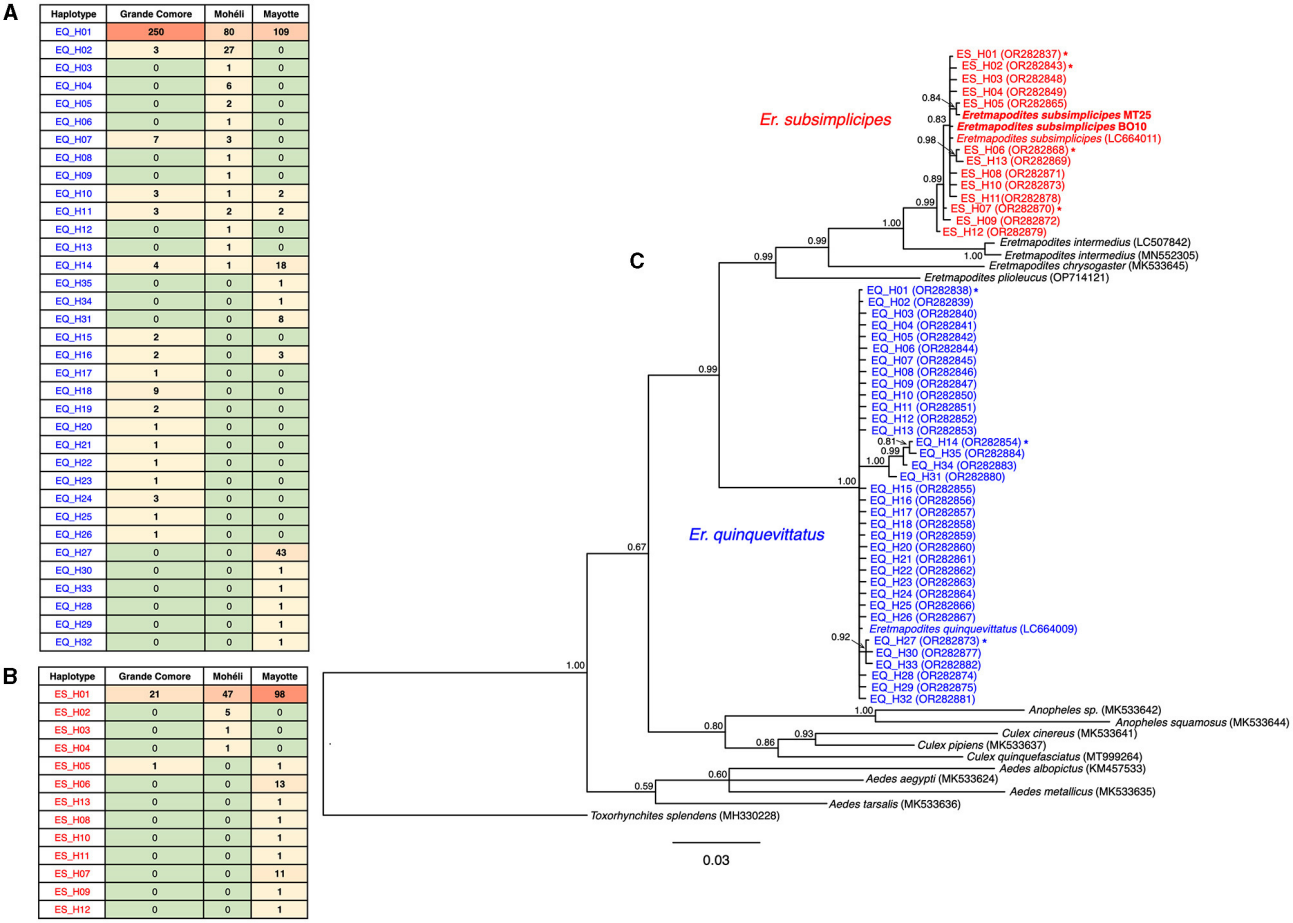
**(A)** Heatmap showing the distribution of *COI* haplotypes in *Eretmapodites quinquevittatus* from three islands of Comoros archipelago; **(B)** Heatmap showing the distribution of the *COI* haplotypes in *Eretmapodites subsimplicipes* from the three surveyed islands; **(C)** Bayesian phylogenetic tree based on *COI gene* (658 bp, 66 sequences) showing relationships between *Er. subsimplicipes* (in red) *Er. quinquevittatus* (in blue) mosquitoes. The phylogenetic tree was built using the substitution model: GTR+I+G. Only unique mtDNA haplotypes are shown: ES_H01 to ES_H13 for *Er. subsimplicipes* and EQ_H01 to EQ_H32 for *Er. quinquevittatus*. Asterisks indicate mitochondrial haplotypes detected in both *Wolbachia* infected and uninfected specimens. Names in bold indicate specimens morphologically identified as *Er. subsimplicipes* in this study. GenBank accession numbers are indicated in brackets. The tree was rooted on *Toxorhynchites splendens* and the numbers associated with nodes indicate the posterior probability. The scale bar is in units of substitutions/site.

Among the 627 *Er. quinquevittatus* specimens, *COI* sequences with good qualities (i.e., 658 bp with no ambiguities) were obtained for 615 samples leading to 35 haplotypes (Figure 2A; Supplementary Table 4). Pairwise nucleotide identity between the haplotypes ranged from 98.2% to 99.9%. The overall haplotype diversity (*Hd*) and nucleotide diversity (π) values were 0.481 and 0.002, respectively. The most frequent haplotype [EQ_H01, found in 71.4% of sequences (*N* = 439/615)] was also the most widespread in all three islands (Figure 2A; Supplementary Table 4). The second most frequent haplotype (EQ_H27, scored in 43 specimens) was geographically restricted to Mayotte (Supplementary Table 4). Of the 35 haplotypes, four haplotypes were shared by all three islands (EQ_H01, EQ_H10, EQ_H11, and EQ_H14), two haplotypes (EQ_H02 and EQ_H07) were shared by Grande Comore and Mohéli, one haplotype (EQ_H16) was common to Grande Comore and Mayotte, and no common haplotype was detected between Mohéli and Mayotte (Supplementary Table 4). On Grande Comore, 18 haplotypes were found whereas the number of haplotypes was similar between Mohéli and Mayotte (14 haplotypes on each island). A total of 11, eight and nine haplotypes were unique on Grande Comore, Mohéli and Mayotte, respectively (Figure 2A; Supplementary Table 4).

*COI* good quality sequences were obtained for 205 of the 252 *Er. subsimplicipes* samples leading to 13 haplotypes (Figure 2B; Supplementary Table 5). Pairwise nucleotide identity between the haplotypes yielded values ranging from 99.4 to 99.9%. The overall *Hd* and π values were 0.338 and 0.001, respectively. The haplotype ES_H01 was the most frequently observed in the dataset, with 80.9% of specimens (*N* = 166/205) and the only one common to all three islands (Figure 2B; Supplementary Table 5). The number of haplotypes was higher in Mayotte (ten haplotypes), followed by Mohéli (four haplotypes), while the lowest diversity was observed in Grande Comore (two haplotypes). Unique haplotypes were only found in Mayotte (eight haplotypes) and Mohéli (three haplotypes) (Figure 2B; Supplementary Table 5).

We assessed phylogenetic relationships between the two *Eretmapodites* species by incorporating the *COI* haplotypes identified in the present study and those of other mosquito species retrieved from GenBank including sequences of *Eremapodites* mosquitoes: *Er. quinquevittatus* (GenBank: LC664009), *Er. subsimplicipes* (GenBank: LC664011), *Eretmapodites intermedius* (GenBank: LC507842 and MN552305), *Eretmapodites chrysogaster* (GenBank: MK533645), and *Eretmapodites plioleucus* (GenBank: OP714121). The phylogenetic tree revealed that *Er. quinquevittatus* and *Er. subsimplicipes* formed two well-supported clades (Figure 2C). Although higher haplotype diversity was found in *Er. quinquevittatus*, the genetic cluster formed by *Er. subsimplicipes* appears slightly more diverged than that of *Er. quinquevittatus*.

### Lower prevalence of *Wolbachia* in *Er. quinquevittatus* than in *Er. subsimplicipes*

The 879 *Eretmapodites* mosquitoes were screened for *Wolbachia* infection based on the detection of the *wsp* gene by PCR. The overall prevalence of *Wolbachia* was 25.7% (*N* = 226/879), with a significant lower prevalence detected in *Er. quinquevittatus* (0.8%, *N* = 5/627) as compared to *Er. subsimplicipes* (87.7%, *N* = 221/252) (Table 1) (Fisher's exact test, *P* < 0.001). *Wolbachia* infections were detected in both males and females of both mosquito species (Table 1). In *Er. quinquevittatus*, two out of the five *Wolbachia* infected mosquitoes were females and three were males. In contrast, the majority of the *Wolbachia* infected *Er. subsimplicipes* mosquitoes were females, with 201 females and 3 males out of a total of 221 infected mosquitoes. For *Er. quinquevittatus*, the bacterial infection prevalence between sites ranged from 0.0% to 7.7% with the five *Wolbachia*-infected specimens detected from five sites: two sites in Grande Comore, one site in Mohéli and two sites in Mayotte (Supplementary Table 1). For *Er. subsimplicipes, Wolbachia*-positive specimens were detected in all but one site (the Iconi site on Grande Comore, *N* = 32 sites with *Er. subsimplicipes* specimens) and infection prevalence ranged from 50.0% to 100.0% including in sites with a large number of samples (Figure 1B; Supplementary Table 1). For both mosquito species, *Wolbachia* infection prevalence did not significantly vary according to the sampled islands (Fisher's exact tests, all *P* > 0.7) (Supplementary Table 1).

**Table 1:** Prevalence of *Wolbachia* in *Eretmapodites quinquevittatus* and *Eretmapodites subsimplicipes* in three islands of the Comoros archipelago, based on presence/absence of the *wsp* gene and according to the sex of mosquitoes.

**Island**	**Sex**	**N**	***Eretmapodites quinquevittatus***	***Eretmapodites subsimplicipes***
			**Prevalence of** ***Wolbachia*** **in %**	**Prevalence of** ***Wolbachia*** **in %**
Grande Comore	All	327	0.7 (2/303)	97.1 (22/24)
	Females	209	0.5 (1/188)	90.5 (19/21)
	Males	118	0.9 (1/115)	100.0 (3/3)
Mohéli	All	195	0.8 (1/131)	85.9 (55/64)
	Females	126	0.0 (0/62)	85.9 (55/64)
	Males	69	1.4 (1/69)	-
Mayotte	All	357	1.0 (2/193)	87.8 (144/164)
	Females	320	0.6 (1/156)	87.8 (144/164)
	Males	37	2.7 (1/37)	-
Total	All	879	0.8 (5/627)	87.7 (221/252)
	Females	655	0.5 (2/406)	87.6 (218/249)
	Males	224	1.4 (3/221)	100.0 (3/3)

*N* = total number of mosquitoes examined.

### Two *Wolbachia* A and B supergroups occurred in *Er. quinquevittatus* and *Er. subsimplicipes*

**Figure 3:**
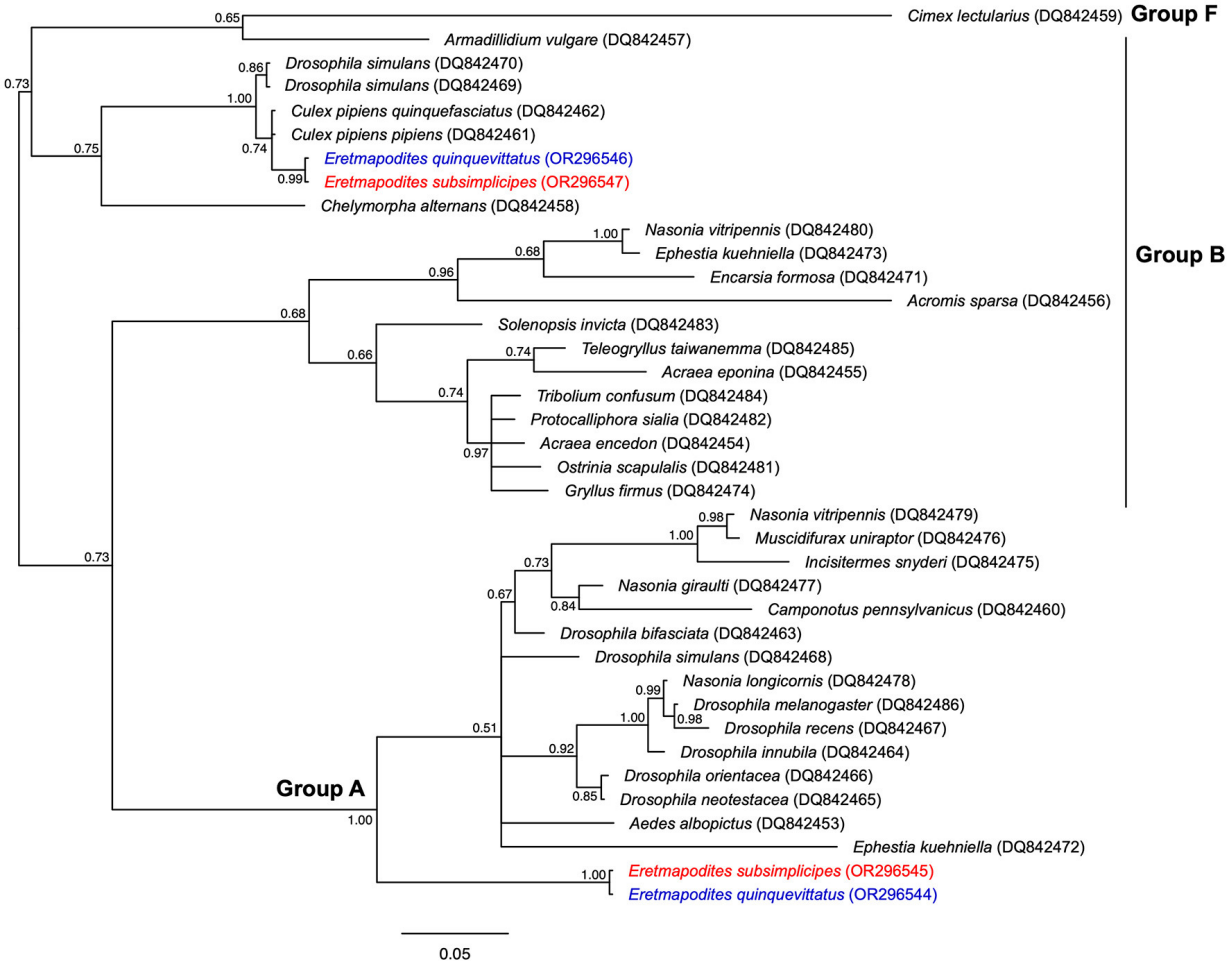
Bayesian phylogenic tree of *Wolbachia* strains based the *wsp* gene (513 bp, 38 sequences). The phylogenetic tree was built using the substitution model: GTR+I+G. Sequences in red and blue correspond to *Wolbachia* sequences detected in *Er. subsimplicipes* and *Er. quinquevittatus*, respectively. The accession numbers of *Wolbachia* strains identified in these mosquito species are also indicated. For each sequence the host species is indicated and the GenBank accession number is provided in brackets. The letters A, B and F correspond to *Wolbachia* supergroups. The tree is midpoint unrooted and the numbers associated with nodes correspond to posterior probability values. The scale bar is in units of substitutions/site.

The sequencing of the *wsp* gene in *Er. quinquevittatus* and *Er. subsimplicipes* revealed the presence of two *Wolbachia* supergroups A and B in each *Eretmapodites* species (Figure 3). For *Er. quinquevittatus*, three samples out of the five *Wolbachia*-infected were successfully sequenced and one sample belonged to supergroup A while two samples belonged to supergroup B (Figure 3; Supplementary Table 3). Concerning *Er. subsimplicipes*, the sequencing of the *wsp* gene was succeful for 218 out of the 221 samples. Almost all of these samples (*N* = 217/218) belonged to supergroup A and one sample to supergroup B. When comparing the 218 *wsp* sequences of supergroup A (217 sequences for *Er. subsimplicipes* and one sequence for *Er. quinquevittatus*), no polymorphism was noted, a unique *wsp* allele shared by the two mosquito species was observed (Figure 3; Supplementary Table 3). The analysis of the three *wsp* sequences from supergroup B (two sequences for *Er. quinquevittatus* and one sequence for *Er. subsimplicipes*) also revealed one *wsp* allele shared by both *Eretmapodites* species.

As the *wsp* gene alone is not relevant for a reliable genotyping of *Wolbachia* strains due to recombination in *Wolbachia* genomes (Jiggins et al., 2001; Bordenstein and Wernegreen, 2004; Baldo et al., 2006; Atyame et al., 2011), we sequenced the five *Wolbachia* MLST genes *coxA, fbpA, ftsZ, gatB* and *hcpA*. The sequences of the five MLST genes were not obtained systematically for each of the 226 *Wolbachia*-infected *Eretmapodites* mosquitoes. Indeed, PCR amplifications have failed for some genes (particularly *fbpA*) in certain samples, possibly due to mutations in the targeted primers sites. Additionally, since we used universal degenerated primers (Baldo et al., 2006), it may have been possible to improve our protocols to increase amplification success for *Eretmapodites*. Ultimately, we obtained 214 sequences for *coxA*, 114 sequences for *fbpA*, 210 sequences for *gatB*, 177 sequences for *hcpA* and 214 sequences for *fstZ*. We confirmed the presence of *Wolbachia* strains belonging to supergroups A and B with each of the five MLST genes (Supplementary Figures 1–5; Supplementary Table 3). We found two alleles for four of the five genes (*coxA, fbpA, ftsZ* and *gatB*), one allele belonging to supergroup A and the other one to supergroup B (Supplementary Figures 1–4; Supplementary Table 3). The most polymorphic locus was *hcpA* with three alleles, two alleles for supergroup A and one allele for supergroup B (Supplementary Figure 5; Supplementary Table 3). The two *hcpA* alleles falling in the supergroup A were genetically close, with 99.8% pairwise identity based on 476 bp. Our data do not support co-infection by *Wolbachia* strains from supergroups A and B. None of the five MLST genes could be amplified in the single *Er. quinquevittatus* sample infected with a *Wolbachia* strain from supergroup A. Therefore, using the MLST genes, we detected supergroup A only in *Er. subsimplicipes* and supergroup B in both *Er. subsimplicipes* and *Er. quinquevittatus* (Supplementary Figures 1–5; Supplementary Table 3). As observed with the *wsp* gene, all MLST alleles were shared by the two mosquito species within supergroup B (Supplementary Figures 1–5).

**Figure 4:**
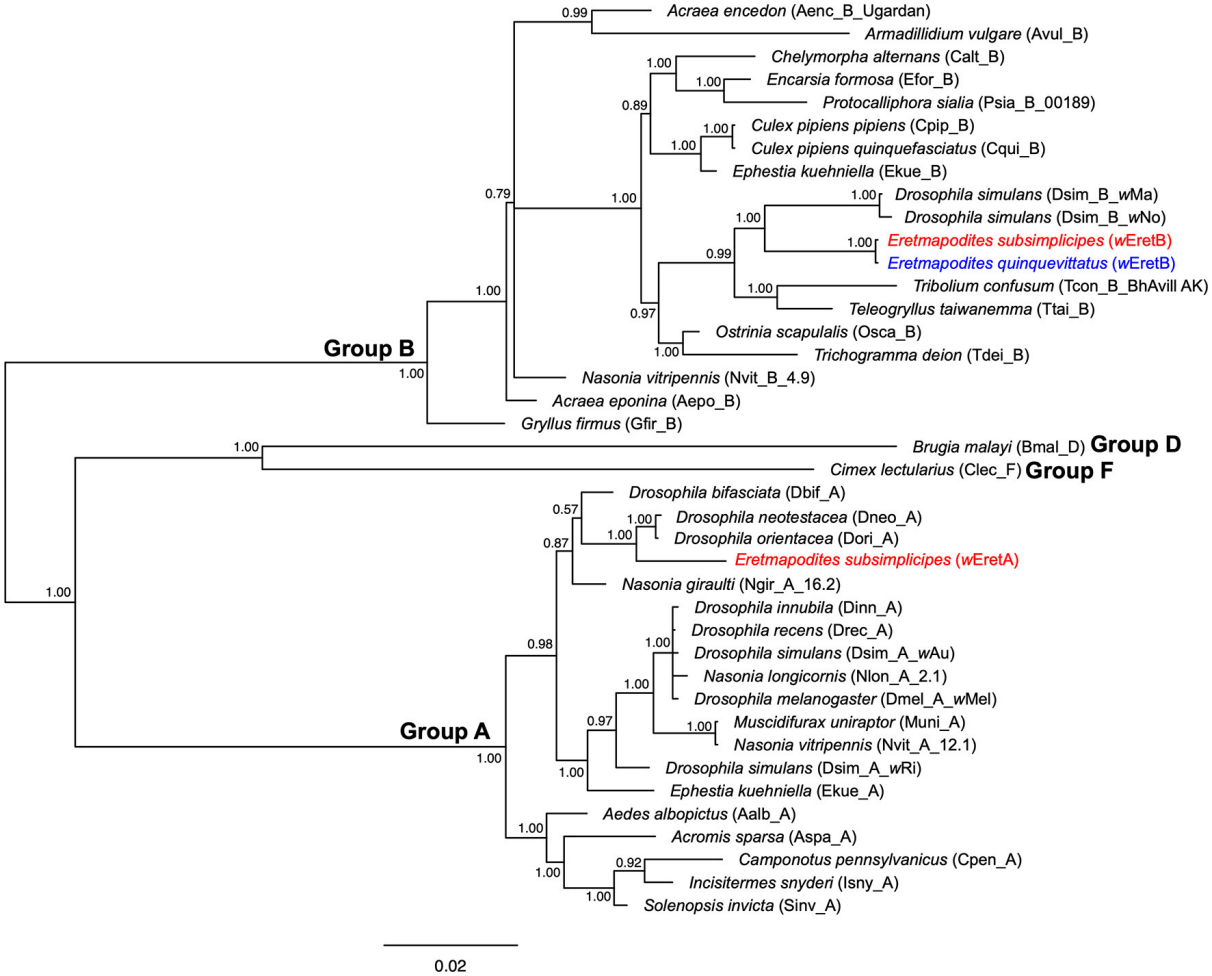
Bayesian phylogenic tree of *Wolbachia* strains obtained from concatenated data set of *MLST* genes (*coxA, fbpA, ftsZ, gatB*, and *hcpA*) (2,079 bp, 40 sequences). The phylogenetic tree was built using the substitution model: GTR+I+G. Sequences in red and blue correspond to *Wolbachia* sequences detected in *Er. subsimplicipes* and *Er. quinquevittatus*, respectively. The names of *Wolbachia* strains identified in these mosquito species (*w*EretA and *w*EretB) are indicated in brackets. The letters A, B, D, and F correspond to *Wolbachia* supergroups. The tree is midpoint unrooted and the numbers associated with nodes correspond to posterior probability values. The scale bar is in units of substitutions/site.

**Figure 5:**
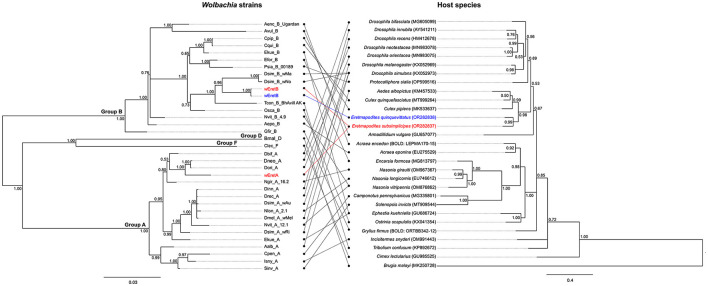
Comparisons between phylogeny of *Wolbachia* and phylogeny of arthropod hosts mitochondria (*COI* gene). The phylogeny of *Wolbachia* was constructed using concatenated sequences of the five MLST genes (*coxA, fbpA, ftsZ, gatB*, and *hcpA*) (2,079 bp, 35 sequences). The mitochondrial phylogeny was constructed using sequences of the *COI* gene (658 bp, 29 sequences). Only *Wolbachia* strains for which the *COI* sequences of the associated host species could be found in public database were used. The letters A, B, D and F correspond to *Wolbachia* supergroups. For *COI* phylogeny, the GenBank or BOLD accession numbers are indicated in brackets. The two phylogenies were built with Bayesian inference using the substitution model: GTR+I+G. The phylogenies were midpoint unrooted and the numbers associated with nodes correspond to posterior probability values. The scale bar is in units of substitutions/site.

Comparison of allelic polymorphism with pubMLST database revealed that within the supergroup A, alleles identified in the present study for *coxA, fbpA, ftsZ*, and *hcpA* are new with the exception of the *gatB* allele matching with allele #49 (Supplementary Table 6). The *coxA* allele showed a close match with allele #173, the *fbpA* allele with allele #60, the *ftsZ* allele with allele #52, and the two *hcpA* alleles were genetically closely related to allele #11 (Supplementary Table 6). The combination of the five alleles resulted in a new *Wolbachia* strain type, which we named “*w*EretA.” For supergroup B, all observed alleles for the five MLST genes are already present in the pubMLST database. Indeed, *coxA, fbpA, ftsZ, gatB*, and *hcpA* alleles matched with alleles #281, #453, #244, #283, and #309, respectively (Supplementary Table 6). However, no *Wolbachia* strain type was assigned to the combination of these five alleles in the pubMLST database. Hence, we considered this *Wolbachia* strain type as new and named it “*w*EretB.” The MLST allelic profiles of *w*EretA and *w*EretB appeared genetically different from those of a *Wolbachia* strain previously described in the species *Eretmapodites chrysogaster* from Cameroon for which *coxA* matched with #275, *ftsZ* matched with #106, and *fbpA* matched with #6 (Osuna et al., 2023). Using complete MLST profiles obtained for 84 mosquitoes (83 *Er. subsimplicipes* and one *Er. quinquevittatus*), we performed a phylogenetic analysis based on the 2,079 bp concatenated sequences of the five MLST genes. It appears that *Wolbachia* strains *w*EretA (infecting *Er. subsimplicipes*) and *w*EretB (infecting both *Er. subsimplicipes* and *Er. quinquevittatus*) form two robust monophyletic clades within A and B supergroups, respectively (Figure 4). *w*EretA is genetically closely related to *w*Dori and *w*Dneo infecting *Drosophila orientacea* and *Drosophila neotestacea*, respectively (Figure 4). *w*EretB is closely related to *w*Ma infecting *Drosophila simulans* (Figure 4). In summary, MLST data revealed that (i) *w*EretA is restricted to *Er. subsimplicipes* (83 complete MLST allelic profiles) and (ii) *w*EretB infects both *Er. subsimplicipes* and *Er. quinquevittatus* (1 complete MLST allelic profile each) (Figure 4; Supplementary Table 3). Finally, we examined the evolution of the diversity of *Wolbachia* in their hosts by comparing the concatenated MLST phylogeny and the *COI* phylogeny from different host species including in *Er. subsimplicipes* and *Er. quinquevittatus*. No congruence between *Wolbachia* and *COI* phylogenies was shown (Figure 5), demonstrating that *Wolbachia* mainly use horizontal transfers to spread in their hosts.

### *Wolbachia* is maternally inherited in *Er. subsimplicipes*

To assess maternal transmission of *Wolbachia* in *Eretmapodites* mosquitoes, we focused on the species *Er. subsimplicipes* as it is the only species for which we currently have a laboratory colony. We examined the progeny of 30 *w*EretA infected laboratory females (see above) based on the sequencing of the *wsp* gene. In general, the number of eggs per female ranging from 4 to 61 (mean number of 32 eggs per female) (Table 2). The hatching rate of the eggs ranged from 5% to 100%, with a mean rate of 74%. It seems that the number of adults produced by each female is limited, as the mean rates for eggs becoming larvae and larvae reaching the adult stage are only 15% and 26%, respectively (Table 2). A total of 131 offspring (74 males and 57 females) from the 30 investigated females were then screened for the presence of *Wolbachia*. Sixty per cent (*N* = 78/131) were found infected (Table 2), leading to a maternal transmission of *Wolbachia* ranging from 0% to 100%. Among the 30 females, four females did not transmit *Wolbachia* to their offspring, the transmission of *Wolbachia* was imperfect (between 6% and 88%) for ten females while perfect maternal transmission of *Wolbachia* (100%) was recorded for 16 females (Table 2).

**Table 2:** Maternal transmission of *Wolbachia* in *Eretmapodites subsimplicipes*.

**Female**	**N eggs**	**N larvae**	**% egg hatching**	**% emergence**	**% egg reaching to adult**	**Adult progeny**						**% maternal transmission**
					**Number of adults**			***Wolbachia*** **positive samples**			
					**Total**	♂	♀	**Total**	♂	♀	
Eret_01	16	16	100%	25%	25%	4	3	1	3	2	1	75%
Eret_02	18	7	39%	43%	17%	3	1	2	2	1	1	67%
Eret_03	21	9	43%	11%	5%	1	1	0	1	1	0	100%
Eret_04	22	20	91%	15%	14%	3	1	2	1	0	1	33%
Eret_05	24	21	88%	5%	4%	1	1	0	0	0	0	0%
Eret_07	51	49	96%	4%	4%	2	0	2	2	0	2	100%
Eret_08	61	60	98%	3%	3%	2	0	2	2	0	2	100%
Eret_09	16	15	94%	7%	6%	1	0	1	1	0	1	100%
Eret_10	4	1	25%	100%	25%	1	1	0	1	1	0	100%
Eret_11	27	6	22%	50%	11%	3	3	0	3	3	0	100%
Eret_12	15	6	40%	17%	7%	1	0	1	1	0	1	100%
Eret_14	39	39	100%	8%	8%	3	0	3	3	0	3	100%
Eret_15	39	30	77%	27%	21%	8	4	4	8	4	4	100%
Eret_16	54	52	96%	8%	7%	4	4	0	4	4	0	100%
Eret_17	38	2	5%	50%	3%	1	0	1	1	0	1	100%
Eret_19	33	18	55%	11%	6%	2	2	0	2	2	0	100%
Eret_20	56	53	95%	2%	2%	1	1	0	1	1	0	100%
Eret_21	39	22	56%	14%	8%	3	3	0	3	3	0	100%
Eret_22	15	15	100%	27%	27%	4	3	1	3	2	1	75%
Eret_23	32	26	81%	65%	53%	17	12	5	1	1	0	6%
Eret_24	55	54	98%	9%	9%	5	2	3	4	1	3	80%
Eret_25	34	33	97%	24%	24%	8	3	5	8	3	5	100%
Eret_26	29	22	76%	36%	28%	8	4	4	7	3	4	88%
Eret_27	10	3	30%	33%	10%	1	0	1	0	0	0	0%
Eret_28	54	46	85%	33%	28%	15	7	8	0	0	0	0%
Eret_29	32	29	91%	21%	19%	6	3	3	0	0	0	0%
Eret_30	43	40	93%	23%	21%	9	4	5	7	2	5	78%
Eret_31	17	10	59%	100%	59%	10	8	2	6	4	2	60%
Eret_32	36	34	94%	6%	6%	2	1	1	1	0	1	50%
Eret_33	34	28	82%	7%	6%	2	2	0	2	2	0	100%
***Mean/Total***		***74%***	***26%***	***15%***	***131***	***74***	***57***	***78***	***40***	***38***	

The presence of *Wolbachia* in females and their offspring was examined through the presence/absence of the *wsp* gene. For each female, the number of eggs, the number of larvae (first instar larvae), the egg hatching rate (%), the percentage of emerging adults, the percentage of egg reaching to adult and the number of emerging adults (males and females) were provided. Finally, the rate of maternal transmission of *Wolbachia* was determined from adult progeny of each female. *N* = total number of mosquitoes examined (eggs or larvae).

## Discussion

Using morphological and molecular methods, we confirmed the presence of two *Eretmapodites* species, *Er. quinquevittatus* and *Er. subsimplicipes*, in three islands of the Comoros archipelago (Grande Comore, Mohéli and Mayotte) (Le Goff et al., 2014; Boussès et al., 2018). The two species occurred in sympatry in the majority of investigated sites but *Er. quinquevittatus* was most commonly found in the three islands. The higher abundance of *Er. quinquevittatus* observed in this study may be due to sampling biases related to the type of samples collected and the method used for collection. In contrast to a previous study conducted between 2008 and 2012 in Mayotte, which found *Er. subsimplicipes* to be the most frequently encountered mosquito species on the island (Le Goff et al., 2014), our observations were based on adult mosquitoes. The difference between our findings and the previous study could be attributed to the fact that larvae were sampled in the study of Le Goff et al. (2014), whereas we focused on adult collection. It is possible that breeding sites of *Er. quinquevittatus* are less accessible compared to those of *Er. subsimplicipes*, which could result in sampling bias when working with adults that have the ability to fly far away from their breeding sites. However, it is also plausible that the distribution area of *Er. quinquevittatus* in Mayotte has increased over the last past 10 years. Additionally, the sampling method used in our study, which involved portable electric aspirators to collect resting adult mosquitoes in vegetation and flying adults around manipulators, may have better suited the collection of *Er. quinquevittatus* adults compared to *Er. subsimplicipes*. Since the biology and ecology of both species in the field are not well understood, it is possible that this methodological difference influenced our findings. It would be interesting in future investigations to compare the distribution area of both *Eretmapodites* species in Mayotte, but also in the other islands of the Comoros archipelago, using both larval and adult sampling.

The mtDNA polymorphism based on the *COI* gene revealed 13 and 35 haplotypes in *Er. subsimplicipes* and *Er. quinquevittatus*, respectively. In both species, we found unique haplotypes (i.e., encountered in only one island), suggesting different colonization events probably from Madagascar or the east coast of Africa, regions geographically close to the Comoros archipelago and where both *Eretmapodites* species have been also identified (Harbach, 2007; Tantely et al., 2016). Other mtDNA haplotypes were shared by different islands and could be the result of a single colonization event of *Eretmapodites* mosquitoes (from Madagascar or Africa) either to different islands, or to one island followed by a secondary dispersion event in a stepping stone mode. Such dispersion from a nearby island can be facilitated by frequent trade between the islands of the Comoros archipelago (Roger et al., 2014). For example, it is well known that the spread of the Asian tiger mosquito *Ae. albopictus* worldwide has been facilitated by the international trade of used tires (Reiter and Sprenger, 1987).

The mitochondrial haplotype diversity was higher in *Er. quinquevittatus* (35 haplotypes, *Hd* = 0.481 and π = 0.002, for *N* = 615 samples) than in *Er. subsimplicipes* (13 haplotypes, *Hd* = 0.338 and π = 0.001, for *N* = 205 samples). The difference between the two species can be explained by the sampling sizes as we found more *Er. quinquevittatus* specimens in our dataset. Alternatively, a higher mtDNA diversity in *Er. quinquevittatus* could result from a low prevalence of *Wolbachia* infection. Indeed, mitochondria and *Wolbachia* are in linkage disequilibrium, both cytoplasmic elements being linked through maternal cotransmission within egg cytoplasm's (Rasgon et al., 2006; Atyame et al., 2011; Dumas et al., 2013). Therefore, the spread of *Wolbachia* in host populations should result in an indirect selective sweep of the mtDNA leading to a reduction of mitochondrial diversity in *Wolbachia* infected host populations (Rasgon et al., 2006; Atyame et al., 2011; Dumas et al., 2013). In this study, we detected *Wolbachia* for the first time in both *Er. quinquevittatus* and *Er. subsimplicipes*, the lowest *Wolbachia* prevalence occurring in *Er. quinquevittatus* (0.8% vs. 87.7% in *Er. subsimplicipes*).

*Wolbachia* infection is not fixed in any of the *Eretmapodites* field populations, with *Wolbachia*-infected and uninfected specimens found within the same sampling sites. The presence of *Wolbachia*-infected and uninfected specimens is commonly observed in field populations of other arthropod species and can be associated with low phenotypic manipulation but also to imperfect maternal transmission (Werren et al., 2008). We have monitored maternal transmission of (the most frequent) *w*EretA using a laboratory colony of *Er. subsimplicipes*. It should be noted that it was challenging to rear *Er. subsimplicipes* species under insectary conditions and a reduced number of adult offspring was obtained for each female. Despite this challenge, our results show that maternal transmission of *Wolbachia* is imperfect or non-existent in some females, which could explain why *Wolbachia* infection is not fixed in *Er. subsimplicipes* field populations. *COI* sequencing data is also consistent with imperfect maternal transmission in *Er. subsimplicipes* since identical mtDNA haplotypes are shared by *Wolbachia*-infected and uninfected mosquitoes (Figure 2C).

The examined phylogenies of *wsp* and each of the five MLST genes showed that both *Er. quinquevittatus* and *Er. subsimplicipes* are infected with two *Wolbachia* supergroups A and B. Within each *Wolbachia* supergroup, the genetic diversity was low, only one allele being detected for almost all loci (except for *hcpA*). The concatenated phylogeny of the five MLST genes also confirmed the presence of two *Wolbachia* supergroups A and B strains (namely *w*EretA and *w*EretB, respectively) in our dataset. In *Er. subsimplicipes*, mosquitoes were infected with either *w*EretA or *w*EretB, although more higher infections by *w*EretA than *w*EretB were observed; while only *w*EretB was observed in *Er. quinquevittatus*. The presence of two divergent *Wolbachia* strains in *Er. subsimplicipes* can be explained by horizontal transfer events from other arhtropod species infected with genetically related *Wolbachia* such as *Drosophila spp*. which appeared to be infected with *Wolbachia* strains closely related to the strains *w*EretA and *w*EretB (Figure 4). Interestingly, the strain *w*EretB was shared by *Er. subsimplicipes* and *Er. quinquevittatus*. Several hypotheses can be proposed to explain this pattern. The *w*EretB strain might have been present in the common ancestor of both *Eretmapodites* species, and this *Wolbachia* strain was maintained in both species after their divergence, but the absence of nucleotide diversity between *w*EretB infecting both mosquito species does not support this assumption. For instance, some difference exists between the *Wolbachia* strains Dinn_A and Drec_A (within the supergroup A) infecting the genetically closely related *Drosphila* species *D. innibula* and *D. recens* (Figure 4). Another possibility would be horizontal transfers of *Wolbachia* between both mosquito species or from other host species. The widespread distribution of *Wolbachia* in arthropods is commonly associated with horizontal transfers occurring between closely related or genetically divergent host species (Heath et al., 1999; Ahmed et al., 2016; Tolley et al., 2019). These transfers would take place through mechanisms such as contamination, predation, or parasitism, particularly among species sharing the same ecological niches. Although evidence of horizontal transfers is rare, studies have shown that such transfers can occur in host-parasitoid associations (Huigens et al., 2004; Ahmed et al., 2015). The lack of congruence between *Wolbachia* and hosts phylogenies also support the possibility of horizontal transfers of *Wolbachia* between species (Tolley et al., 2019). In our study, we compared the phylogenies of concatenated MLST genes and *COI*, and no congruence was found (Figure 5), confirming the potential for horizontal transfers of *Wolbachia* between host species. Furthermore, the success of interspecific transfers of *Wolbachia* via embryonic microinjections (Sasaki and Ishikawa, 2000; McMeniman et al., 2009; Hughes and Rasgon, 2014) also supports the hypothesis of horizontal transfers of *Wolbachia*. Assuming a horizontal transfer of *w*EretB from *Er. subsimplicipes* to *Er. quinquevittatus*, the low prevalence of *Wolbachia* in *Er. quinquevittatus* could be explained either by a recent transfer of *w*EretB, or by differences in phenotypes induced by this *Wolbachia* strain when infecting each mosquito species. This change in phenotype expression of the same *Wolbachia* strain when infecting different host species has been previously described in *Drosophila recens* and *Drosophila subquinaria* (Jaenike, 2007). It would be interesting for future investigations to examine the phenotypes induced by the *Wolbachia* strains *w*EretA and *w*EretB in *Er. subsimplicipes* and *Er. quinquevittatus* to better understand the dynamics of these bacteria in the field. Lastly, a horizontal transfer of *w*EretB might have happened through introgression between both *Eretmapodites* species. Introgressions of *Wolbachia* have been observed in various subspecies of mosquitoes in the *Culex pipiens* complex, such as *Culex pipiens pipiens* and *Culex pipiens quinquefasciatus*, which hybridize in natural environments. This hybridization leads to subspecies sharing the same *Wolbachia* strains, as determined through the *Wolbachia* MLST genotyping method (Atyame et al., 2011; Dumas et al., 2013) (see also Figure 4). This hypothesis could be tested in the future by comparing the polymorphism in nuclear genomes of *Er. subsimplicipes* and *Er. quinquevittatus* mosquitoes.

## Conclusion

In the present study, we characterized the mitochondrial genetic diversity of *Er. quinquevittatus* and *Er. subsimplicipes* occurring in sympatry in three islands of the Comoros archipelago. We also characterized the genetic diversity of *Wolbachia* infecting both mosquito species and identified two new *Wolbachia* strains, which have been named *w*EretA and *w*EretB. Experimental rearing of *Er. subsimplicipes* revealed imperfect maternal transmission of *Wolbachia* that might explain the infection patterns found in the field. Future studies will examine the phenotypes induced by these *Wolbachia* in *Er. quinquevittatus* and *Er. subsimplicipes* to better understand their dynamics *in natura*. As *Eretmapodites* mosquitoes are competent vectors for the transmission of arboviruses (Bamou et al., 2021; Cêtre-Sossah et al., 2023), future investigations should also consider the effects of *w*EretA and *w*EretB on vector competence.

## Data availability statement

The datasets presented in this study can be found in online repositories. The names of the repository/repositories and accession number(s) can be found below: https://www.ncbi.nlm.nih.gov/genbank/, OR282837-OR282884, OR296528-OR296530, OR296531-OR296533, OR296534-OR296536, OR296537-OR296539, OR296540-OR296543, and OR296544-OR296547.

## Ethics statement

The manuscript presents research on animals that do not require ethical approval for their study.

## Author contributions

YG: Data curation, Formal analysis, Methodology, Writing—original draft, Writing—review & editing. SH: Conceptualization, Methodology, Writing—original draft, Writing—review & editing. CL: Investigation, Methodology, Writing—original draft. PR: Investigation, Resources, Writing—original draft. A-bI: Investigation, Resources, Writing—original draft. AY: Investigation, Resources, Writing—original draft. PB: Formal analysis, Methodology, Writing—original draft. PM: Conceptualization, Funding acquisition, Validation, Writing—review & editing. CA: Conceptualization, Investigation, Methodology, Supervision, Validation, Writing—original draft, Writing—review & editing.

## References

[B1] Ahmed M. Z. Breinholt J. W. Kawahara A. Y. (2016). Evidence for common horizontal transmission of *Wolbachia* among butterflies and moths. BMC Evolut. Biol. 16:118. 10.1186/s12862-016-0660-x 27233666 PMC4882834

[B2] Ahmed M. Z. Li S.-J. Xue X. Yin X.-J. Ren S.-X. Jiggins F. M.. (2015). The intracellular bacterium *Wolbachia* uses parasitoid wasps as phoretic vectors for efficient horizontal transmission. PLoS Pathog. 10:e1004672. 10.1371/journal.ppat.1004672 25675099 PMC4347858

[B3] Aliota M. T. Peinado S. A. Velez I. D. Osorio J. E. (2016). The *w*Mel strain of *Wolbachia* reduces transmission of Zika virus by *Aedes aegypti*. Sci. Rep. 6:28792. 10.1038/srep28792 27364935 PMC4929456

[B4] Armbruster P. Damsky W. E. Jr. Giordano R. Birungi J. Munstermann L. E. Conn J. E. (2003). Infection of new- and old-world *Aedes albopictus* (Diptera: *Culicidae*) by the intracellular parasite *Wolbachia*: implications for host mitochondrial DNA evolution. J. Med. Entomol. 40, 356–360. 10.1603/0022-2585-40.3.356 12943116

[B5] Atyame C. M. Delsuc F. Pasteur N. Weill M. Duron O. (2011). Diversification of *Wolbachia* endosymbiont in the *Culex pipiens* mosquito. Molec. Biol. Evolut. 28, 2761–2772. 10.1093/molbev/msr083 21515811

[B6] Atyame C. M. Labbé P. Dumas E. Milesi P. Charlat S. Fort P.. (2014). *Wolbachia* divergence and the evolution of cytoplasmic incompatibility in *Culex pipiens*. PLoS ONE 9:e87336. 10.1371/journal.pone.0087336 24498078 PMC3909092

[B7] Ayala D. Akone-Ella O. Rahola N. Kengne P. Ngangue M. F. Mezeme F.. (2019). Natural *Wolbachia* infections are common in the major malaria vectors in Central Africa. Evolut. Applic. 12, 1583–1594. 10.1111/eva.12804 31462916 PMC6708434

[B8] Baldini F. Segata N. Pompon J. Marcenac P. Robert Shaw W. Dabiré R. K.. (2014). Evidence of natural *Wolbachia* infections in field populations of *Anopheles gambiae*. Natu. Commun. 5:3985. 10.1038/ncomms4985 24905191 PMC4059924

[B9] Baldo L. Dunning Hotopp J. C. Jolley K. A. Bordenstein S. R. Biber S. A. Choudhury R. R.. (2006). Multilocus sequence typing system for the endosymbiont *Wolbachia pipientis*. Appl. Environ. Microbiol. 72, 7098–7110. 10.1128/AEM.00731-06 16936055 PMC1636189

[B10] Bamou R. Mayi M. P. A. Djiappi-Tchamen B. Nana-Ndjangwo S. M. Nchoutpouen E. Cornel A. J.. (2021). An update on the mosquito fauna and mosquito-borne diseases distribution in Cameroon. Paras. Vectors 14:527. 10.1186/s13071-021-04950-9 34635176 PMC8507310

[B11] Bauer J. H. (1928). The transmission of yellow fever by mosquitoes other than *Aedes aegypti*. J. Am. Med. Assoc. 90, 2091–2092. 10.1001/jama.1928.02690530019007

[B12] Bian G. Joshi D. Dong Y. Lu P. Zhou G. Pan X.. (2013). *Wolbachia* Invades *Anopheles stephensi* populations and induces refractoriness to *Plasmodium* infection. Science 340, 748–751. 10.1126/science.1236192 23661760

[B13] Bian G. Xu Y. Lu P. Xie Y. Xi Z. (2010). The endosymbiotic bacterium *Wolbachia* induces resistance to dengue virus in *Aedes aegypti*. PLOS Pathog. 6:e1000833. 10.1371/journal.ppat.1000833 20368968 PMC2848556

[B14] Bordenstein S. R. Paraskevopoulos C. Dunning Hotopp J. C. Sapountzis P. Lo N. Bandi C.. (2009). Parasitism and mutualism in *Wolbachia*: what the phylogenomic trees can and cannot say. Molec. Biol. Evol. 26, 231–241. 10.1093/molbev/msn243 18974066 PMC2721558

[B15] Bordenstein S. R. Wernegreen J. J. (2004). Bacteriophage flux in endosymbionts (*Wolbachia*): infection frequency, lateral transfer, and recombination rates. Molec. Biol. Evolut. 21, 1981–1991. 10.1093/molbev/msh211 15254259

[B16] Bourtzis K. Dobson S. L. Xi Z. Rasgon J. L. Calvitti M. Moreira L. A.. (2014). Harnessing mosquito–*Wolbachia* symbiosis for vector and disease control. Acta Tropica 132, S150–S163. 10.1016/j.actatropica.2013.11.004 24252486

[B17] Boussès P. Le Goff G. Robert V. (2018). Inventaire des moustiques (Diptera : *Culicidae*) des îles du sud-ouest de l'océan Indien, Madagascar excepté — Une revue critique. Ann. Soc. Entomol. France 54, 89–110. 10.1080/00379271.2018.1429951

[B18] Braig H. R. Zhou W. Dobson S. L. O'Neill S. L. (1998). Cloning and characterization of a gene encoding the major surface protein of the bacterial endosymbiont *Wolbachia pipientis*. J. Bacteriol. 180, 2373–2378. 10.1128/JB.180.9.2373-2378.1998 9573188 PMC107178

[B19] Cêtre-Sossah C. Lebon C. Rabarison P. Cardinale E. Mavingui P. Atyame C. (2023). Evidence of *Eretmapodites subsimplicipes* and *Aedes albopictus* as competent vectors for Rift Valley fever virus transmission in Mayotte. Acta Tropica 239:106835. 10.1016/j.actatropica.2023.106835 36649804

[B20] Coon K. L. Brown M. R. Strand M. R. (2016). Mosquitoes host communities of bacteria that are essential for development but vary greatly between local habitats. Molec. Ecol. 25, 5806–5826. 10.1111/mec.13877 27718295 PMC5118126

[B21] Darriba D. Taboada G. L. Doallo R. Posada D. (2012). jModelTest 2: more models, new heuristics and parallel computing. Nat. Methods 9, 772–772. 10.1038/nmeth.2109 22847109 PMC4594756

[B22] Dodson B. L. Hughes G. L. Paul O. Matacchiero A. C. Kramer L. D. Rasgon J. L. (2014). *Wolbachia* enhances West Nile Virus (WNV) infection in the mosquito *Culex tarsalis*. PLoS Negl. Trop. Dis. 8:e2965. 10.1371/journal.pntd.0002965 25010200 PMC4091933

[B23] Douglas A. E. (1998). Nutritional interactions in insect-microbial symbioses: aphids and their symbiotic bacteria *Buchnera*. Ann. Rev. Entomol. 43, 17–37. 10.1146/annurev.ento.43.1.17 15012383

[B24] Dumas E. Atyame C. M. Milesi P. Fonseca D. M. Shaikevich E. V. Unal S.. (2013). Population structure of *Wolbachia* and cytoplasmic introgression in a complex of mosquito species. BMC Evolut. Biol. 13:181. 10.1186/1471-2148-13-181 24006922 PMC3846486

[B25] Duron O. Bouchon D. Boutin S. Bellamy L. Zhou L. Engelstädter J.. (2008). The diversity of reproductive parasites among arthropods: *Wolbachia* do not walk alone. BMC Biol. 6:27. 10.1186/1741-7007-6-27 18577218 PMC2492848

[B26] Duron O. Fort P. Weill M. (2005). Hypervariable prophage WO sequences describe an unexpected high number of *Wolbachia* variants in the mosquito *Culex pipiens*. Proc. R. Soc. B. 273, 495–502. 10.1098/rspb.2005.3336 16615218 PMC1560203

[B27] Dutra H. L. C. Rocha M. N. Dias F. B. S. Mansur S. B. Caragata E. P. Moreira L. A. (2016). *Wolbachia* blocks currently circulating Zika Virus isolates in Brazilian *Aedes aegypti* mosquitoes. Cell Host Micr. 19, 771–774. 10.1016/j.chom.2016.04.021 27156023 PMC4906366

[B28] Edwards F. W. (1941). Mosquitoes of the Ethiopian Region. III.-Culicine aults and pupae. Available online at: https://www.cabdirect.org/cabdirect/abstract/19411000211 (accessed November 2, 2023).

[B29] Folmer O. Black M. Hoeh W. Lutz R. Vrijenhoek R. (1994). DNA primers for amplification of mitochondrial cytochrome coxidase subunit-I from diverse metazoan invertebrates. Molec. Marine Biol. Biotechnol. 15, 294–299. 7881515

[B30] Glowska E. Dragun-Damian A. Dabert M. Gerth M. (2015). New *Wolbachia* supergroups detected in quill mites (Acari: Syringophilidae). Infect. Genet. Evolut. 30, 140–146. 10.1016/j.meegid.2014.12.019 25541519

[B31] Harbach R. E. (2007). The Culicidae (Diptera): a review of taxonomy, classification and phylogeny. Zootaxa 1668, 591–638. 10.11646/zootaxa.1668.1.28

[B32] Harbach R. E. (2013). Mosquito taxonomic inventory. Available online at: https://mosquito-taxonomic-inventory.myspecies.info/how-reference-site (accessed November 3, 2023).

[B33] Heath B. D. Butcher R. D. J. Whitfield W. G. F. Hubbard S. F. (1999). Horizontal transfer of *Wolbachia* between phylogenetically distant insect species by a naturally occurring mechanism. Curr. Biol. 9, 313–316. 10.1016/S0960-9822(99)80139-0 10209097

[B34] Hedges L. M. Brownlie J. C. O'Neill S. L. Johnson K. N. (2008). *Wolbachia* and virus protection in insects. Science 322, 702–702. 10.1126/science.1162418 18974344

[B35] Hilgenboecker K. Hammerstein P. Schlattmann P. Telschow A. Werren J. H. (2008). How many species are infected with *Wolbachia*? – a statistical analysis of current data. FEMS Microbiol. Lett. 281, 215–220. 10.1111/j.1574-6968.2008.01110.x 18312577 PMC2327208

[B36] Hoffmann A. A. Montgomery B. L. Popovici J. Iturbe-Ormaetxe I. Johnson P. H. Muzzi F.. (2011). Successful establishment of *Wolbachia* in *Aedes* populations to suppress dengue transmission. Nature 476, 454–457. 10.1038/nature10356 21866160

[B37] Hopkins G. H. E. (1952). Mosquitoes of the Ethiopian Region. I. Larval bionomics of mosquitoes and taxonomy of Culicine larvae. Available online at: https://www.cabdirect.org/cabdirect/abstract/19532901687 (accessed November 2, 2023).

[B38] Hughes G. L. Rasgon J. L. (2014). Transinfection: a method to investigate *Wolbachia*-host interactions and control arthropod-borne disease. Insect Molec. Biol. 23, 141–151. 10.1111/imb.12066 24329998 PMC3949162

[B39] Hughes G. L. Vega-Rodriguez J. Xue P. Rasgon J. L. (2012). *Wolbachia* strain *w*AlbB enhances infection by the rodent malaria parasite *Plasmodium berghei* in *Anopheles gambiae* mosquitoes. Appl. Environ. Microbiol. 78, 1491–1495. 10.1128/AEM.06751-11 22210220 PMC3294472

[B40] Huigens M. E. de Almeida R. P. Boons P.a,. H Luck R. F. Stouthamer R. (2004). Natural interspecific and intraspecific horizontal transfer of parthenogenesis-inducing *Wolbachia* in *Trichogramma* wasps. Proc. R. Soc. B. 271, 509–515. 10.1098/rspb.2003.2640 15129961 PMC1691627

[B41] Jaenike J. (2007). Spontaneous emergence of a new *Wolbachia* phenotype. Evolution 61, 2244–2252. 10.1111/j.1558-5646.2007.00180.x 17767593

[B42] Jaenike J. Unckless R. Cockburn S. N. Boelio L. M. Perlman S. J. (2010). Adaptation via symbiosis: recent spread of a *Drosophila* defensive symbiont. Science 329, 212–215. 10.1126/science.1188235 20616278

[B43] Jiggins F. M. Schulenburg J. H. G. von der Hurst, G. D. D. Majerus M. E. N. (2001). Recombination confounds interpretations of *Wolbachia* evolution. Proc. R. Soc. B. 268, 1423–1427. 10.1098/rspb.2001.1656 11429144 PMC1088758

[B44] Jolley K. A. Bray J. E. Maiden M. C. J. (2018). Open-access bacterial population genomics: BIGSdb software, the PubMLST.org website and their applications. Wellcome Open Res. 3:124. 10.12688/wellcomeopenres.14826.1 30345391 PMC6192448

[B45] Kambhampati S. Rai K. S. Burgun S. J. (1993). Unidirectional cytoplasmic incompatibility in the mosquito *Aedes albopictus*. Evolution 47, 673–677. 10.2307/2410079 28568710

[B46] Kearse M. Moir R. Wilson A. Stones-Havas S. Cheung M. Sturrock S.. (2012). Geneious Basic: an integrated and extendable desktop software platform for the organization and analysis of sequence data. Bioinformatics 28, 1647–1649. 10.1093/bioinformatics/bts199 22543367 PMC3371832

[B47] Laven H. (1951). Crossing experiments with *Culex* strains. Evolution 5, 370–375. 10.2307/2405682

[B48] Le Goff G. Goodman S. M. Elguero E. Robert V. (2014). Survey of the mosquitoes (Diptera: *Culicidae*) of Mayotte. PLoS ONE 9:e100696. 10.1371/journal.pone.0100696 25004163 PMC4086827

[B49] McIntosh B. M. Jupp P.G. Dos Santos I. Barnard B.J.H. (1980). Vector studies on Rift Valley Fever Virus in South Africa. South African Med. J. 58, 127–132. 6105722

[B50] McMeniman C. J. Lane R. V. Cass B. N. Fong A. W. C. Sidhu M. Wang Y.-F.. (2009). Stable introduction of a life-shortening *Wolbachia* infection into the mosquito *Aedes aegypti*. Science 323, 141–144. 10.1126/science.1165326 19119237

[B51] Moreira L. A. Iturbe-Ormaetxe I. Jeffery J. A. Lu G. Pyke A. T. Hedges L. M.. (2009). A *Wolbachia* symbiont in *Aedes aegypti* limits infection with Dengue, Chikungunya, and *Plasmodium*. Cell 139, 1268–1278. 10.1016/j.cell.2009.11.042 20064373

[B52] Musa A. A. Muturi M. W. Musyoki A. M. Ouso D. O. Oundo J. W. Makhulu E. E.. (2020). Arboviruses and blood meal sources in zoophilic mosquitoes at human-wildlife interfaces in Kenya. Vector-Borne Zoonotic Dis. 20, 444–453. 10.1089/vbz.2019.2563 32155389

[B53] Oliver K. M. Russell J. A. Moran N. A. Hunter M. S. (2003). Facultative bacterial symbionts in aphids confer resistance to parasitic wasps. Proc. Natl. Acad. Sci. 100, 1803–1807. 10.1073/pnas.0335320100 12563031 PMC149914

[B54] Osuna A. M. Gidley A. Mayi M. P. A. Bamou R. Dhokiya V. Antonio-Nkondjio C.. (2023). Diverse novel *Wolbachia* bacteria strains and genera-specific co-infections with *Asaia* bacteria in *Culicine* mosquitoes from ecologically diverse regions of Cameroon. Wellcome Open Res. 8:267. 10.12688/wellcomeopenres.18580.2 37799509 PMC10548110

[B55] Paraskevopoulos C. Bordenstein S. R. Wernegreen J. J. Werren J. H. Bourtzis K. (2006). Toward a *Wolbachia* multilocus sequence typing system: discrimination of *Wolbachia* strains present in *Drosophila* species. Curr.Microbiol. 53, 388–395. 10.1007/s00284-006-0054-1 17036209

[B56] Rambaut A. (2014). FigTree 1.4.2 software.

[B57] Rasgon J. L. Cornel A. J. Scott T. W. (2006). Evolutionary history of a mosquito endosymbiont revealed through mitochondrial hitchhiking. Proc. R. Soc. B. 273, 1603–1611. 10.1098/rspb.2006.3493 16769630 PMC1634923

[B58] Reiter P. Sprenger D. (1987). The used tire trade: a mechanism for the worldwide dispersal of container breeding mosquitoes. J. Am. Mosquito Control Assoc. 3, 494–501. 2904963

[B59] Roger M. Beral M. Licciardi S. Soulé M. Faharoudine A. Foray C.. (2014). Evidence for circulation of the rift valley fever virus among livestock in the union of comoros. PLoS Negl. Trop. Dis. 8:e3045. 10.1371/journal.pntd.0003045 25078616 PMC4117442

[B60] Ronquist F. Teslenko M. van der Mark P. Ayres D. L. Darling A. Höhna S.. (2012). MrBayes 3.2: efficient bayesian phylogenetic inference and model choice across a large model space. System. Biol. 61, 539–542. 10.1093/sysbio/sys029 22357727 PMC3329765

[B61] Ros V. I. D. Fleming V. M. Feil E. J. Breeuwer J. A. J. (2009). How diverse is the genus *Wolbachia*? Multiple-gene sequencing reveals a putatively new *Wolbachia* supergroup recovered from spider mites (Acari: *Tetranychidae*). Appl. Environ. Microbiol. 75, 1036–1043. 10.1128/AEM.01109-08 19098217 PMC2643572

[B62] Rozas J. Ferrer-Mata A. Sánchez-DelBarrio J. C. Guirao-Rico S. Librado P. Ramos-Onsins S. E.. (2017). DnaSP 6: DNA sequence polymorphism analysis of large data sets. Molec. Biol. Evol. 34, 3299–3302. 10.1093/molbev/msx248 29029172

[B63] Sasaki T. Ishikawa H. (2000). Transinfection of *Wolbachia* in the mediterranean flour moth, *Ephestia kuehniella*, by embryonic microinjection. Heredity 85, 130–135. 10.1046/j.1365-2540.2000.00734.x 11012714

[B64] Scarborough C. L. Ferrari J. Godfray H. C. J. (2005). Aphid protected from pathogen by endosymbiont. Science 310, 1781–1781. 10.1126/science.1120180 16357252

[B65] Service M. W. (1990). Handbook to the Afrotropical toxorhynchitine and culicine mosquitoes, excepting Aedes and Culex. London: British Museum (Natural History) 1–207.

[B66] Shropshire J. D. Leigh B. Bordenstein S. R. (2020). Symbiont-mediated cytoplasmic incompatibility: what have we learned in 50 years? eLife 9:e61989. 10.7554/eLife.61989 32975515 PMC7518888

[B67] Sicard M. Bonneau M. Weill M. (2019). *Wolbachia* prevalence, diversity, and ability to induce cytoplasmic incompatibility in mosquitoes. Curr. Opin. Insect Sci. 34, 12–20. 10.1016/j.cois.2019.02.005 31247412

[B68] Tantely M. L. Goff G. L. Boyer S. Fontenille D. (2016). An updated checklist of mosquito species (Diptera: Culicidae) from Madagascar. Parasite 23:20. 10.1051/parasite/2016018 27101839 PMC4840257

[B69] Teixeira L. Ferreira Á. Ashburner M. (2008). The bacterial symbiont *Wolbachia* induces resistance to RNA viral infections in *Drosophila melanogaster*. PLoS Biol. 6:e1000002. 10.1371/journal.pbio.1000002 19222304 PMC2605931

[B70] Thongsripong P. Chandler J. A. Green A. B. Kittayapong P. Wilcox B. A. Kapan D. D.. (2018). Mosquito vector-associated microbiota: metabarcoding bacteria and eukaryotic symbionts across habitat types in Thailand endemic for dengue and other arthropod-borne diseases. Ecol. Evolut. 8, 1352–1368. 10.1002/ece3.3676 29375803 PMC5773340

[B71] Tokash-Peters A. G. Niyonzima J. D. Kayirangwa M. Muhayimana S. Tokash I. W. Jabon J. D.. (2022). Mosquito microbiomes of Rwanda: characterizing mosquito Host and microbial communities in the land of a thousand hills. bioRxiv 2022.08.03.502589. 10.1101/2022.08.03.502589 PMC11062966 38691215

[B72] Tolley S. J. A. Nonacs P. Sapountzis P. (2019). *Wolbachia* horizontal transmission events in ants: what do we know and what can we learn? Front. Microbiol. 10:296. 10.3389/fmicb.2019.00296 30894837 PMC6414450

[B73] Tortosa P. Charlat S. Labbé P. Dehecq J.-S. Barré H. Weill M. (2010). *Wolbachia* age-sex-specific density in *Aedes albopictus*: a host evolutionary response to cytoplasmic incompatibility? PLoS ONE 5:e9700. 10.1371/journal.pone.0009700 20300514 PMC2838780

[B74] Tsuchida T. Koga R. Horikawa M. Tsunoda T. Maoka T. Matsumoto S.. (2010). Symbiotic bacterium modifies aphid body color. Science 330, 1102–1104. 10.1126/science.1195463 21097935

[B75] Turelli M. Katznelson A. Ginsberg P. S. (2022). Why *Wolbachia*-induced cytoplasmic incompatibility is so common. Proc. Natl. Acad. Sci. 119:e2211637119. 10.1073/pnas.2211637119 36343219 PMC9704703

[B76] Walker T. Johnson P. H. Moreira L. A. Iturbe-Ormaetxe I. Frentiu F. D. McMeniman C. J.. (2011). The *w*Mel *Wolbachia* strain blocks dengue and invades caged *Aedes aegypti* populations. Nature 476, 450–453. 10.1038/nature10355 21866159

[B77] Weinert L. A. Araujo-Jnr E. V. Ahmed M. Z. Welch J. J. (2015). The incidence of bacterial endosymbionts in terrestrial arthropods. Proc. R. Soc. B. 282:20150249. 10.1098/rspb.2015.0249 25904667 PMC4424649

[B78] Werren J. H. Baldo L. Clark M. E. (2008). *Wolbachia*: master manipulators of invertebrate biology. Nat. Rev. Microbiol. 6, 741–751. 10.1038/nrmicro1969 18794912

[B79] Zélé F. Nicot A. Berthomieu A. Weill M. Duron O. Rivero A. (2014). *Wolbachia* increases susceptibility to *Plasmodium* infection in a natural system. Proc. R. Soc. B. 281:20132837. 10.1098/rspb.2013.2837 PMC3924077 24500167

[B80] Zhou W. Rousset F. O'Neil S. (1998). Phylogeny and PCR-based classification of *Wolbachia* strains using *wsp* gene sequences. Proc. R. Soc. B. 265, 509–515. 10.1098/rspb.1998.0324 9569669 PMC1688917

[B81] Zouache K. Raharimalala F. N. Raquin V. Tran-Van V. Raveloson L. H. R. Ravelonandro P.. (2011). Bacterial diversity of field-caught mosquitoes, *Aedes albopictus* and *Aedes aegypti*, from different geographic regions of Madagascar. FEMS Microbiol. Ecol. 75, 377–389. 10.1111/j.1574-6941.2010.01012.x 21175696

[B82] Zug R. Hammerstein P. (2012). Still a host of hosts for *Wolbachia*: analysis of recent data suggests that 40% of terrestrial arthropod species are infected. PLoS ONE 7:e38544. 10.1371/journal.pone.0038544 22685581 PMC3369835

