# Erratum: Genetic diversity of endosymbiotic bacteria *Wolbachia* infecting two mosquito species of the genus *Eretmapodites* occurring in sympatry in the Comoros archipelago

**DOI:** 10.3389/fmicb.2024.1425304

**Published:** 2024-05-09

**Authors:** Frontiers Production Office

**Affiliations:** Frontiers Media SA, Lausanne, Switzerland

**Keywords:** *Wolbachia*, *Eretmapodites quinquevittatus*, *Eretmapodites subsimplicipes*, mitochondrial genetic diversity, Comoros archipelago

Due to a production error, the affiliation of Editor SK was incorrectly formatted. The affiliation is Tata Institute for Genetics and Society, India.

The publisher apologizes for this mistake. The original article has been updated.

