# Supplementary material for: Genetic diversity of endosymbiotic bacteria Wolbachia infecting two mosquito species of the genus Eretmapodites occurring in sympatry in the Comoros archipelago
Source: Front Microbiol. 2024 Mar 27;15:1343917. doi: 10.3389/fmicb.2024.1343917 (PMC11004463; doi:10.3389/fmicb.2024.1343917)
Supplement: Supplementary file 2 [file Data_Sheet_1.doc]

**Supplementary Information**

**Genetic diversity of endosymbiotic bacteria *Wolbachia* infecting two mosquito species of the genus *Eretmapodites* occurring in sympatry in the islands of the Comoros archipelago**

Yann Gomard^1‡†^, Sarah Hafsia^1‡^, Cyrille Lebon^1^, Patrick Rabarison^2^, Ambdoul-bar Idaroussi^2^, Amina Yssouf^3^, Philippe Boussès^4^, Patrick Mavingui^1*^ and Célestine Atyame^1*^

**Supplementary Tables**

**Table 1. Prevalence of *Wolbachia* in *Eretmapodites* *quinquevittatus* and *Eretmapodites* *subsimplicipes* populations in three islands of the Comoros archipelago: Grande Comore, Mohéli and Mayotte.**

| **Island** | **Site** | **Date** | **Coordinates** | | **N** | ***Er. quinquevittatus*** | |  | ***Er. subsimplicipes*** | |
| --- | --- | --- | --- | --- | --- | --- | --- | --- | --- | --- |
|  |  |  | **Latitude** | **Longitude** |  | **N1** | **Prevalence of *Wolbachia* infection (%) (n1)** |  | **N2** | **Prevalence of *Wolbachia* infection (%) (n2)** |
| *Grande Comore* | Bahani | 29/11/19 | -11.6415449 | 43.2930221 | 77 | 69 | 0.0 (0) |  | 8 | 100.0 (8) |
|  | Iconi | 29/11/19 | -11.7352600 | 43.2401963 | 24 | 23 | 0.0 (0) |  | 1 | 0.0 (0) |
|  | Koimbani | 02/12/19 | -11.6159581 | 43.3594671 | 26 | 26 | 0.0 (0) |  | - | - |
|  | Mbambani | 29/11/19 | -11.8510460 | 43.3386146 | 8 | 8 | 0.0 (0) |  | - | - |
|  | Mirontsi | 03/12/19 | -11.6809223 | 43.2726607 | 1 | 1 | 0.0 (0) |  | - | - |
|  | Mitsoudjé | 29/11/19 | -11.8038464 | 43.2769982 | 21 | 15 | 0.0 (0) |  | 6 | 100.0 (6) |
|  | Moindzazadjumbe | 29/11/19 | -11.7711357 | 43.2620878 | 6 | 3 | 0.0 (0) |  | 3 | 66.7 (2) |
|  | Moroni _1 | 27/11/19 | -11.6992647 | 43.2520399 | 74 | 72 | 1.4 (1) |  | 2 | 100.0 (2) |
|  | Moroni _3 | 27/11/19 | -11.7046200 | 43.2546777 | 14 | 14 | 0.0 (0) |  | - | - |
|  | Moroni_4 | 27/11/19 | -11.7114796 | 43.2466336 | 11 | 11 | 0.0 (0) |  | - | - |
|  | Moroni_5 | 27/11/19 | -11.7246173 | 43.2489390 | 3 | 3 | 0.0 (0) |  | - | - |
|  | Moroni_6 | 28/11/19 | -11.7138374 | 43.2535718 | 14 | 12 | 0.0 (0) |  | 2 | 100.0 (2) |
|  | Moroni_7 | 28/11/19 | -11.7211027 | 43.2401785 | 13 | 13 | 7.7 (1) |  | - | - |
|  | Moroni_8 | 30/11/19 | -11.7203296 | 43.2508396 | 14 | 14 | 0.0 (0) |  | - | - |
|  | Moroni_9 | 03/12/19 | -11.6894313 | 43.2654218 | 12 | 12 | 0.0 (0) |  | - | - |
|  | Singani | 29/11/19 | -11.8469161 | 43.3116024 | 6 | 4 | 0.0 (0) |  | 2 | 100.0 (2) |
|  | Vouvoni | 28/11/19 | -11.7557711 | 43.2486859 | 1 | 1 | 0.0 (0) |  | - | - |
|  | Vouvoni_2 | 30/11/19 | -11.7573690 | 43.2553159 | 2 | 2 | 0.0 (0) |  | - | - |
|  | ***Total*** |  |  |  | ***327*** | ***303*** | ***0.7 (2)*** |  | ***24*** | ***91.7 (22)*** |

**Table 1 continued**

| **Island** | **Site** | **Date** | **Coordinates** | | **N** | ***Er. quinquevittatus*** | |  | | ***Er. subsimplicipes*** | | |
| --- | --- | --- | --- | --- | --- | --- | --- | --- | --- | --- | --- | --- |
|  |  |  | **Latitude** | **Longitude** |  | **N1** | **Prevalence of *Wolbachia* infection (%) (n1)** | |  | | **N2** | **Prevalence of *Wolbachia* infection (%) (n2)** |
| *Mohéli* | Barakani | 22/11/19 | -12.2865274 | 43.6332385 | 18 | 15 | 0.0 (0) | |  | | 3 | 66.7 (2) |
|  | Hamba | 22/11/19 | -12.2747301 | 43.6347710 | 75 | 35 | 0.0 (0) | |  | | 40 | 87.5 (35) |
|  | Mbouni_Foungue | 21/11/19 | -12.3557090 | 43.7005204 | 14 | 9 | 0.0 (0) | |  | | 5 | 80.0 (4) |
|  | Mbouni_Riviere | 22/11/19 | -12.3534227 | 43.6992051 | 54 | 44 | 0.0 (0) | |  | | 10 | 90.0 (9) |
|  | Miringoni | 21/11/19 | -12.3033814 | 43.6352119 | 22 | 19 | 5.3 (1) | |  | | 3 | 66.7 (2) |
|  | Moheli_Est | 21/11/19 | ND | ND | 6 | 4 | 0.0 (0) | |  | | 2 | 100.0 (2) |
|  | Ouallah | 22/11/19 | -12.3378857 | 43.6690718 | 2 | 2 | 0.0 (0) | |  | | - | - |
|  | Vanilla_lodge | 21/11/19 | -12.3569630 | 43.7152190 | 4 | 3 | 0.0 (0) | |  | | 1 | 100.0 (1) |
|  | ***Total*** |  |  |  | ***195*** | ***131*** | ***0.8 (1)*** | |  | | ***64*** | ***85.9 (55)*** |
| *Mayotte* | Bambo Est | 07/03/19 | -12.9316111 | 45.1757222 | 6 | - | 0.0 (0) | |  | | 6 | 100.0 (6) |
|  | Choungi | 07/03/19 | -12.9630000 | 45.1274722 | 9 | 3 | 0.0 (0) | |  | | 6 | 83.3 (5) |
|  | Combani_1 | 05/03/19 | -12.7796389 | 45.1420555 | 15 | 9 | 0.0 (0) | |  | | 6 | 83.3 (5) |
|  | Combani_2 | 05/03/19 | -12.7848611 | 45.1370833 | 10 | 8 | 0.0 (0) | |  | | 2 | 100.0 (2) |
|  | Dembéni de Sada | 07/03/19 | -12.8429722 | 45.1715277 | 3 | 3 | 0.0 (0) | |  | | - | - |
|  | Doujani_1 | 01/05/19 | -12.7927500 | 45.2099166 | 22 | 20 | 5.0 (1) | |  | | 2 | 50.0 (1) |
|  | Dzoumognié | 06/03/19 | -12.7203611 | 45.1171111 | 3 | 3 | 0.0 (0) | |  | | - | - |
|  | Kahani_1 | 05/03/19  and  02/05/19 | -12.8275833 | 45.1311666 | 25 | 17 | 5.9 (1) | |  | | 8 | 100.0 (8) |
|  | Kahani_2 | 05/03/19 | -12.8296389 | 45.1285833 | 30 | 6 | 0.0 (0) | |  | | 24 | 100.0 (24) |
|  | Kavani | 01/05/19 | -12.7875278 | 45.2226666 | 14 | 14 | 0.0 (0) | |  | | - | - |
|  | Kaweni_3 | 29/04/19 | -12.7618056 | 45.2258333 | 4 | 4 | 0.0 (0) | |  | | - | - |
|  | Kaweni_4 | 29/04/19 | -12.7582778 | 45.2310277 | 5 | 5 | 0.0 (0) | |  | | - | - |
|  | Kaweni_5 | 29/04/19 | -12.7556944 | 45.2268888 | 7 | 7 | 0.0 (0) | |  | | - | - |

**Table 1 continued**

| **Island** | **Site** | **Date** | **Coordinates** | | **N** | ***Er. quinquevittatus*** | |  | ***Er. subsimplicipes*** | |
| --- | --- | --- | --- | --- | --- | --- | --- | --- | --- | --- |
|  |  |  | **Latitude** | **Longitude** |  | **N1** | **Prevalence of *Wolbachia* infection (%) (n1)** |  | **N2** | **Prevalence of *Wolbachia* infection (%) (n2)** |
| *Mayotte* | Mamoudzou_1 | 08/03/19  and 30/04/19 | -12.7823056 | 45.2338611 | 7 | 5 | 0.0 (0) |  | 2 | 100.0 (2) |
|  | Mamoudzou_2 | 08/03/19 | -12.7800000 | 45.2264722 | 1 | 1 | 0.0 (0) |  | - | - |
|  | Mamoudzou_3 | 30/04/19 | -12.7812778 | 45.2237500 | 4 | 3 | 0.0 (0) |  | 1 | 100.0 (1) |
|  | Mamoudzou_4 | 30/04/19 | -12.7803611 | 45.2237500 | 14 | 13 | 0.0 (0) |  | 1 | 100.0 (1) |
|  | Mamoudzou_5 | 30/4/19 | -12.7815556 | 45.2193333 | 2 | 2 | 0.0 (0) |  | - | - |
|  | Mirereni_c1 | 02/05/19 | -12.7902222 | 45.1427222 | 5 | 3 | 0.0 (0) |  | 2 | 100.0 (2) |
|  | Mirereni_c2 | 02/05/19 | -12.7870556 | 45.1400000 | 6 | 5 | 0.0 (0) |  | 1 | 100.0 (1) |
|  | Mtsahara | 06/03/19 | -12.6840278 | 45.0833888 | 7 | 4 | 0.0 (0) |  | 3 | 66.7 (2) |
|  | M'tsangamouji | 03/05/19 | -12.7649167 | 45.0794722 | 11 | 7 | 0.0 (0) |  | 4 | 75.0 (3) |
|  | Passamainty_1 | 01/05/19 | -12.7964167 | 45.2151388 | 8 | 8 | 0.0 (0) |  | - | - |
|  | Poroani | 07/03/19 | -12.8910000 | 45.1391111 | 15 | 12 | 0.0 (0) |  | 3 | 100.0 (3) |
|  | Sada | 03/05/19 | -12.8442222 | 45.1083333 | 36 | 9 | 0.0 (0) |  | 27 | 85.2 (23) |
|  | Tsoundzou_1 | 01/05/19 | -12.8091944 | 45.2026944 | 7 | 1 | 0.0 (0) |  | 6 | 100.0 (6) |
|  | Tsingoni | 03/05/19 | -12.7882222 | 45.1080555 | 1 | 1 | 0.0 (0) |  | - | - |
|  | Vahibe | 02/05/19 | -12.7847222 | 45.1751944 | 80 | 20 | 0.0 (0) |  | 60 | 81.7 (49) |
|  | ***Total*** |  |  |  | ***357*** | ***193*** | ***1.0 (2)*** |  | ***164*** | ***87.8 (144)*** |

Mosquitoes were sampled as adults and were identified by sequencing the mitochondrial *COI* gene. The presence of *Wolbachia* was determined through the presence/absence of the *wsp* gene. For each site, the date of sampling and coordinates are provided. Sites in yellow are those where the two *Eretmapodites* species are in sympatry; sites in grey and green are those where only *Er. quinquevittatus* and only *Er. subsimplicipes* was collected, respectively. N total = total number of mosquitoes examined; N1 = number of mosquitoes belonging to the species *Er. quinquevittatus*; N2 = number of mosquitoes belonging to the species *Er. subsimplicipes*; n1 = number of *Er. quinquevittatus* mosquitoes infected with *Wolbachia*; n2 = number of *Er. subsimplicipes* mosquitoes infected with *Wolbachia*.

**Table 2**. List of primers used to examine the genetic diversity of the two mosquito species *Eretmapodites* *quinquevittatus* and *Eretmapodites subsimplicipes* and the detected *Wolbachia.*

| **Origin** | **Gene** | **Primer (5’ – 3’)** | **PCR annealing temperature** | **Sequence size** | **Reference** |
| --- | --- | --- | --- | --- | --- |
| Mitochondrial gene of mosquito | *COI* | LCO1490 (GGTCAACAAATCATAAAGATATTGG) | 52°C | 658 bp | Folmer et al. (1994) |
|  |  | HCO2198 (TAAACTTCAGGGTGACCAAAAAATCA) |  |  |  |
|  |  |  |  |  |  |
| *Wolbachia* gene | *wsp* | 81F (TGGTCCAATAAGTGATGAAGAAAC) | 53°C | 558 / 579 bp | Braig et al. (1998) |
|  |  | 691R (AAAAATTAAACGCTACTCCA) |  |  |  |
|  |  |  |  |  |  |
|  | *coxA* | coxA_F1 (TTGGRGCRATYAACTTTATAG) | 55°C | 446 bp | Baldo et al. (2006) |
|  |  | coxA_R1 (CTAAAGACTTTKACRCCAGT) |  |  |  |
|  |  |  |  |  |  |
|  | *fbpA* | fbpA_F1 (GCTGCTCCRCTTGGYWTGAT) | 59°C | 465 / 471 bp | Baldo et al. (2006) |
|  |  | fbpA_R1 (CCRCCAGARAAAAYYACTATTC) |  |  |  |
|  |  |  |  |  |  |
|  | *ftsZ* | ftsZuniF (ATYATGGARCATATAAARGATAG) | 55°C | 672 bp | Baldo et al. (2006) |
|  |  | ftsZuniR (TCRAGYAATGGATTRGATAT) |  |  |  |
|  |  |  |  |  |  |
|  | *gatB* | gatB_F1 (GAKTTAAAYCGYGCAGGBGTT) | 57°C | 429 bp | Baldo et al. (2006) |
|  |  | gatB_R1 (TGGYAAYTCRGGYAAAGATGA) |  |  |  |
|  |  |  |  |  |  |
|  | *hcpA* | hcpA_F1 (GAAATARCAGTTGCTGCAAA) | 59°C | 482 bp | Baldo et al. (2006) |
|  |  | hcpA_R1 (GAAAGTYRAGCAAGYTCTG) |  |  |  |

The sequence sizes correspond to the size after the cleaning of sequences.

**Table 3**. Details on all investigated *Eretmapodites* specimens. All specimens were clearly identified based on *COI* sequences but the exact assignation of the haplotype was not possible for all mosquitoes given the presence of ambiguities with sequences and/or short sequences for some samples (*see* Excel file).

**Table 4**. Mitochondrial *COI* haplotypes in 615 *Eretmapodites quinquevittatus* mosquitoes from Grande Comore, Mohéli and Mayotte. Only polymorphic positions are indicated. All haplotypes are compared with the most frequently detected (EQ_H01) that was arbitrarily chosen as reference. Dots correspond to identity with the reference sequence. N indicates the total number of samples with the same haplotype.

**Table 5**. Mitochondrial *COI* haplotypes in 205 *Eretmapodites subsimplicipes* mosquitoes from Grande Comore, Mohéli and Mayotte. Only polymorphic positions are indicated. All haplotypes are compared with the most frequently detected (ES_H01) that was arbitrarily chosen as the reference. Dots correspond to identity with the reference sequence. N indicates the total number of samples with the same haplotype.

| **Haplotype** | **ID of mosquito** | **Position in sequence** | | | | | | | | | | | |  |  | **Island** | | |
| --- | --- | --- | --- | --- | --- | --- | --- | --- | --- | --- | --- | --- | --- | --- | --- | --- | --- | --- |
|  |  | 50 | 122 | 181 | 200 | 220 | 316 | 317 | 368 | 373 | 529 | 616 | 634 |  | N | Grande Comore | Mohéli | Mayotte |
| ES_H01 | ERET_COM_1 | C | G | T | G | T | A | G | G | A | C | C | C |  | 166 | 21 | 47 | 98 |
| ES_H02 | ERET_COM_27 | . | A | . | . | . | . | . | . | . | . | . | . |  | 5 | - | 5 | - |
| ES_H03 | ERET_COM_77 | . | . | . | . | . | . | . | . | G | . | . | . |  | 1 | - | 1 | - |
| ES_H04 | ERET_COM_86 | . | . | . | . | . | . | . | . | . | . | . | T |  | 1 | - | 1 | - |
| ES_H05 | ERET_COM_398 | . | . | . | . | . | . | . | . | . | T | . | . |  | 2 | 1 | - | 1 |
| ES_H06 | VME_5 | . | . | . | . | . | G | . | . | . | . | . | . |  | 13 | - | - | 13 |
| ES_H07 | VME_32 | . | . | . | . | C | . | . | . | . | . | . | . |  | 11 | - | - | 11 |
| ES_H08 | VME_37 | . | . | . | . | . | . | . | A | . | . | . | . |  | 1 | - | - | 1 |
| ES_H09 | VME_49 | . | . | . | A | C | . | . | . | . | . | . | . |  | 1 | - | - | 1 |
| ES_H10 | VME_103 | . | . | . | . | . | . | . | . | . | . | T | . |  | 1 | - | - | 1 |
| ES_H11 | VME_122 | T | . | . | . | . | . | . | . | . | . | . | . |  | 1 | - | - | 1 |
| ES_H12 | VME_134 | . | . | A | . | C | . | . | . | . | . | . | . |  | 1 | - | - | 1 |
| ES_H13 | VME_26 | . | . | . | . | . | G | A | . | . | . | . | . |  | 1 | - | - | 1 |

**Table 6.** Comparisons between alleles of the five MLST genes (*coxA, fbpA, ftsZ, gatB and hcpA*) identified in *Eretmapodites* mosquitoes in this study and alleles from the *Wolbachia* pubMLST database. For comparison with pubMLST database, the number of nucleotide differences are indicated in parentheses when a closest match was obtained.

| ***Wolbachia* supergroup** | ***Wolbachia* strain** | **MLST genes** | **Number of  identified alleles** | **GenBank accession numbers** | **Comparison with pubMLST (number of nucleotide differences)** | **New allele (Yes / No)** |
| --- | --- | --- | --- | --- | --- | --- |
| A | *w*EretA | coxA | 1 | OR296528 | closest match: #173 (1 diff.) | Yes |
|  |  | *fbpA* | 1 | OR296531 | closest match: #60 (4 diff.) | Yes |
|  |  | *ftsZ* | 1 | OR296534 | closest match: #52 (6 diff.) | Yes |
|  |  | *gatB* | 1 | OR296537 | exact macth: #49 | No |
|  |  | *hcpA* | 2 | OR296540 and OR296542 | closest match: #11 (7 and 8 diff) | Yes |
| B | *w*EretB | *coxA* | 1 | OR296529 or OR296530 | exact macth: #281 | No |
|  |  | *fbpA* | 1 | OR296532 or OR296533 | exact macth: #453 | No |
|  |  | *ftsZ* | 1 | OR296535 or OR296536 | exact macth: #244 | No |
|  |  | *gatB* | 1 | OR296538 or OR296539 | exact macth: #283 | No |
|  |  | *hcpA* | 1 | OR296541 or OR296543 | exact macth: #309 | No |

**Supplementary Figures**

**Figure 1. Bayesian phylogenic tree of *Wolbachia* strains based the *coxA* gene (402 bp, 40 sequences).** The phylogenetic tree was built using the substitution model: HKY+I+G. Sequences in red and blue correspond to *Wolbachia* sequences detected in *Er. subsimplicipes* and *Er. quinquevittatus*, respectively. For each sequence the host species is indicated and the GenBank accession number is provided in brackets. The letters A, B, D and F correspond to *Wolbachia* supergroups. The tree is midpoint unrooted and the numbers associated with nodes correspond to posterior probability values. The scale bar is in units of substitutions/site.

**Figure 2. Bayesian phylogenic tree of *Wolbachia* strains based the *fbpA* gene (429 bp, 40 sequences).** The phylogenetic tree was built using the substitution model: HKY+G. Sequences in red and blue correspond to *Wolbachia* sequences detected in *Er. subsimplicipes* and *Er. quinquevittatus*, respectively. For each sequence the host species is indicated and the GenBank accession number is provided in brackets. The letters A, B, D and F correspond to *Wolbachia* supergroups. The tree is midpoint unrooted and the numbers associated with nodes correspond to posterior probability values. The scale bar is in units of substitutions/site.

**Figure 3. Bayesian phylogenic tree of *Wolbachia* strains based the *ftsZ* gene (435 bp, 40 sequences).** The phylogenetic tree was built using the substitution model: GTR+G. Sequences in red and blue correspond to *Wolbachia* sequences detected in *Er. subsimplicipes* and *Er. quinquevittatus*, respectively. For each sequence the host species is indicated and the GenBank accession number is provided in brackets. The letters A, B, D and F correspond to *Wolbachia* supergroups. The tree is midpoint unrooted and the numbers associated with nodes correspond to posterior probability values. The scale bar is in units of substitutions/site.

**Figure 4. Bayesian phylogenic tree of *Wolbachia* strains based the *gatB* gene (369 bp, 40 sequences). taxa and substitution model: GTR+I+G).** The phylogenetic tree was built using the substitution model: GTR+I+G. Sequences in red and blue correspond to *Wolbachia* sequences detected in *Er. subsimplicipes* and *Er. quinquevittatus*, respectively. For each sequence the host species is indicated and the GenBank accession number is provided in brackets. The letters A, B, D and F correspond to *Wolbachia* supergroups. The tree is midpoint unrooted and the numbers associated with nodes correspond to posterior probability values. The scale bar is in units of substitutions/site.

**Figure 5. Bayesian phylogenic tree of *Wolbachia* strains based the *hcpA* gene (444 bp, 41 sequences).** The phylogenetic tree was built using the substitution model: GTR+G. Sequences in red and blue correspond to *Wolbachia* sequences detected in *Er. subsimplicipes* and *Er. quinquevittatus*, respectively. For each sequence the host species is indicated and the GenBank accession number is provided in brackets. The letters A, B, D and F correspond to *Wolbachia* supergroups. The tree is midpoint unrooted and the numbers associated with nodes correspond to posterior probability values. The scale bar is in units of substitutions/site.
