# Supplementary figures and images for: Genetic diversity of endosymbiotic bacteria Wolbachia infecting two mosquito species of the genus Eretmapodites occurring in sympatry in the Comoros archipelago
Source: Front Microbiol. 2024 Mar 27;15:1343917. doi: 10.3389/fmicb.2024.1343917 (PMC11004463; doi:10.3389/fmicb.2024.1343917)

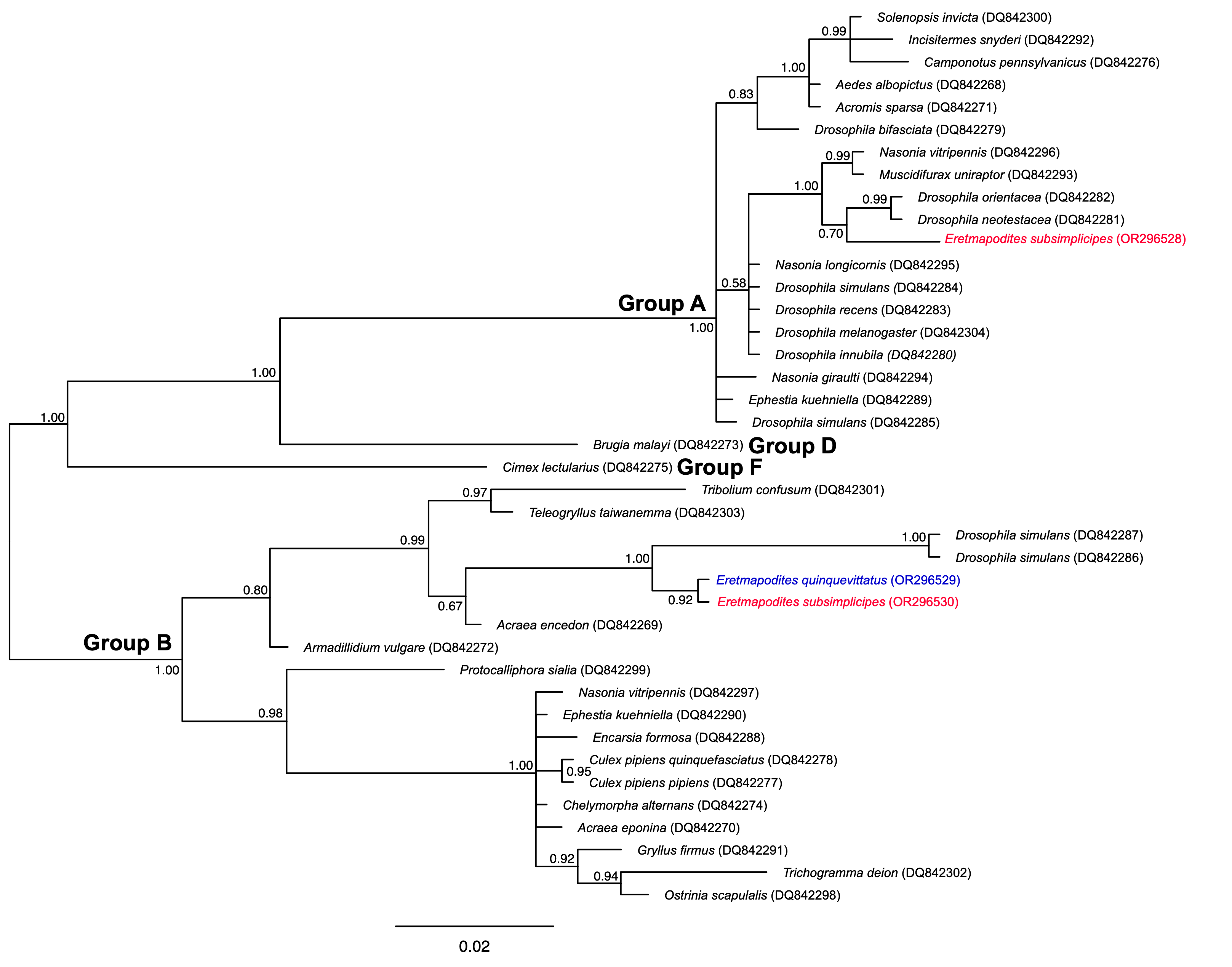

Supplement: Supplementary file 3 [file Image_1.TIFF]

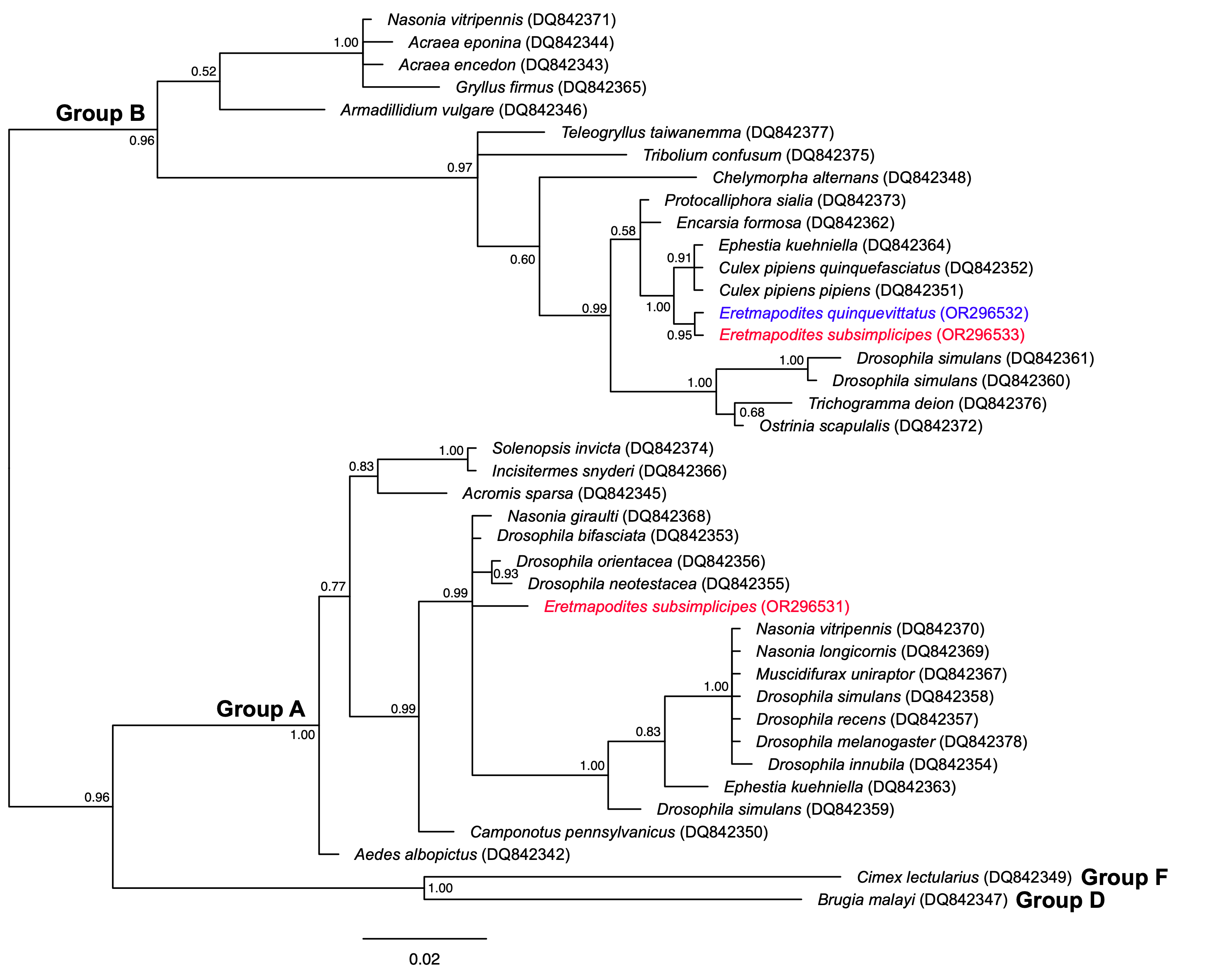

Supplement: Supplementary file 4 [file Image_2.TIFF]

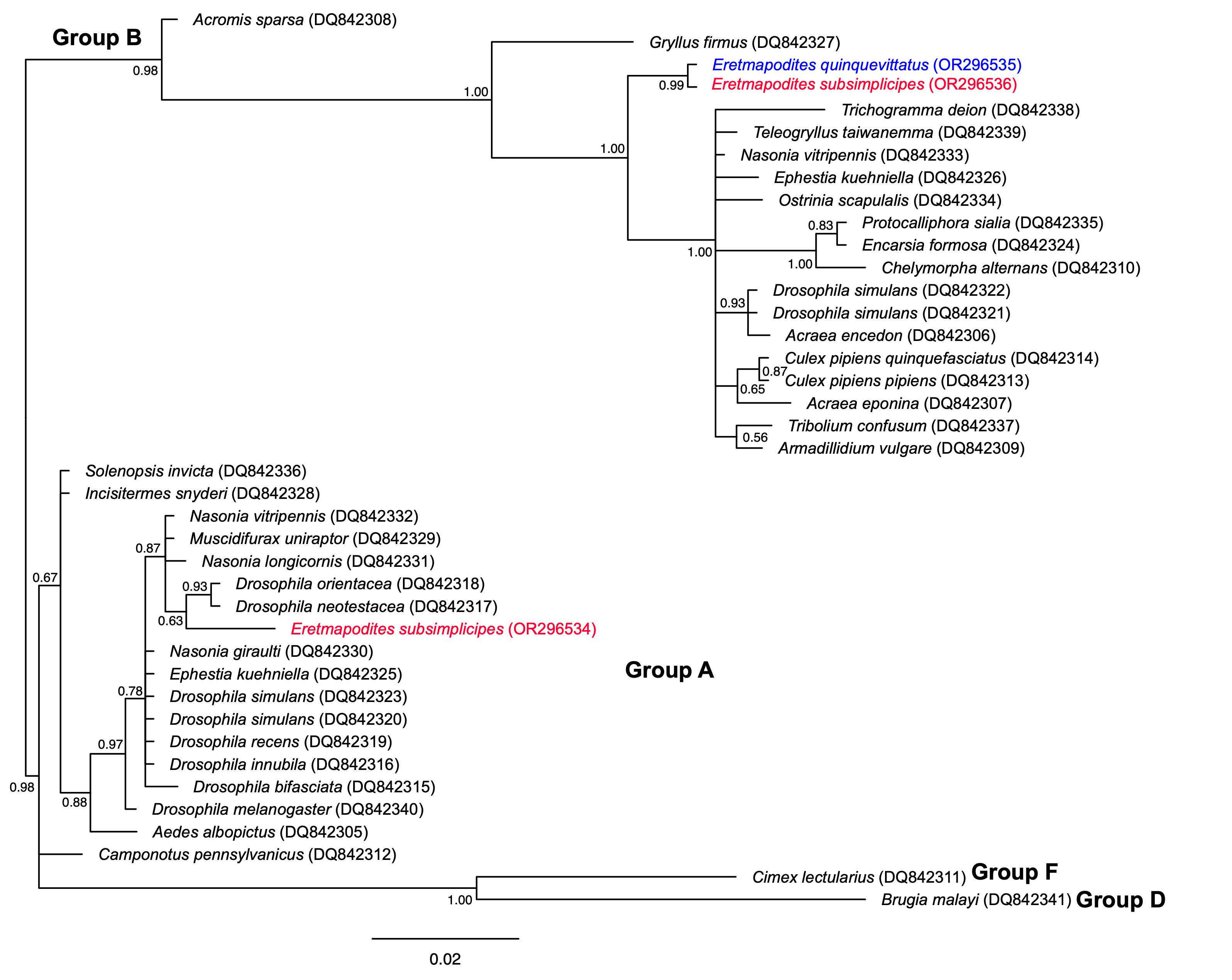

Supplement: Supplementary file 5 [file Image_3.TIFF]

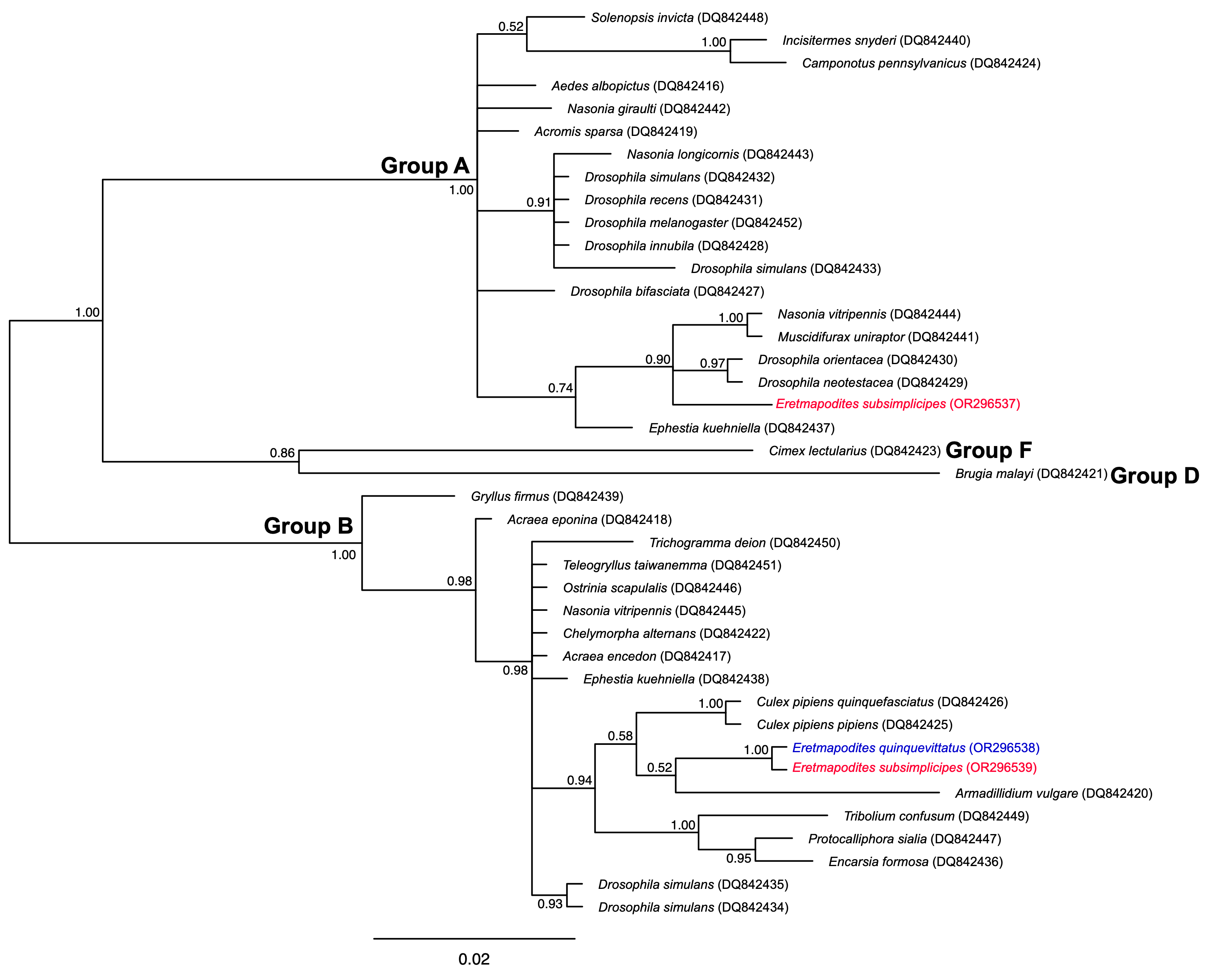

Supplement: Supplementary file 6 [file Image_4.TIFF]

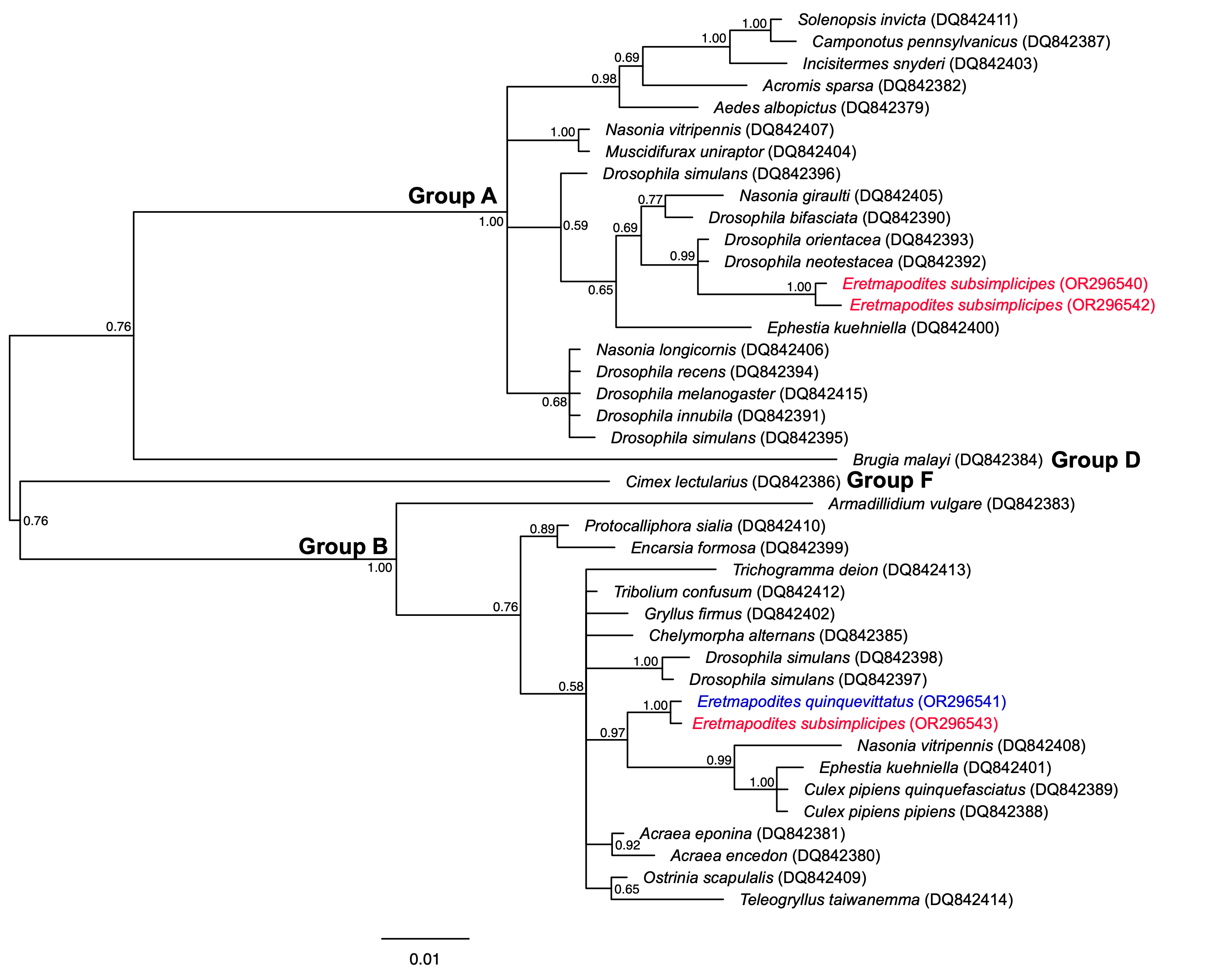

Supplement: Supplementary file 7 [file Image_5.TIFF]
